# TEA domain transcription factor 1 (TEAD1) induces cardiac fibroblasts cells remodeling through BRD4/Wnt4 pathway

**DOI:** 10.1038/s41392-023-01732-w

**Published:** 2024-02-19

**Authors:** Shuai Song, Xiaokai Zhang, Zihang Huang, Yongchao Zhao, Shuyang Lu, Linqi Zeng, Fengze Cai, Tongyao Wang, Zhiqiang Pei, Xinyu Weng, Wei Luo, Hao Lu, Zilun Wei, Jian Wu, Peng Yu, Li Shen, Xiaochun Zhang, Aijun Sun, Junbo Ge

**Affiliations:** 1 Department of Cardiology, Zhongshan Hospital, Fudan University, Shanghai Institute of Cardiovascular Diseases, Shanghai, China; 2 State Key Laboratory of Cardiology, Zhongshan Hospital, Fudan University, Shanghai, China; 3 Key Laboratory of Viral Heart Diseases, National Health Commission, Shanghai, China; 4 Key Laboratory of Viral Heart Diseases, Chinese Academy of Medical Sciences, Shanghai, China; 5 National Clinical Research Center for Interventional Medicine, Shanghai, China; 6 Department of cardiac surgery, Zhongshan Hospital, Fudan University, Shanghai, China; 7 Institutes of Biomedical Sciences, Fudan University, Shanghai, China; 8 Shanghai Institute of Hypertension, Ruijin Hospital, Shanghai Jiao Tong University School of Medicine, Shanghai, China

**Keywords:** Cardiology, Diseases

## Abstract

Cardiac fibroblasts (CFs) are the primary cells tasked with depositing and remodeling collagen and significantly associated with heart failure (HF). TEAD1 has been shown to be essential for heart development and homeostasis. However, fibroblast endogenous TEAD1 in cardiac remodeling remains incompletely understood. Transcriptomic analyses revealed consistently upregulated cardiac TEAD1 expression in mice 4 weeks after transverse aortic constriction (TAC) and Ang-II infusion. Further investigation revealed that CFs were the primary cell type expressing elevated TEAD1 levels in response to pressure overload. Conditional TEAD1 knockout was achieved by crossing TEAD1-floxed mice with CFs- and myofibroblasts-specific Cre mice. Echocardiographic and histological analyses demonstrated that CFs- and myofibroblasts-specific TEAD1 deficiency and treatment with TEAD1 inhibitor, VT103, ameliorated TAC-induced cardiac remodeling. Mechanistically, RNA-seq and ChIP-seq analysis identified Wnt4 as a novel TEAD1 target. TEAD1 has been shown to promote the fibroblast-to-myofibroblast transition through the Wnt signalling pathway, and genetic Wnt4 knockdown inhibited the pro-transformation phenotype in CFs with TEAD1 overexpression. Furthermore, co-immunoprecipitation combined with mass spectrometry, chromatin immunoprecipitation, and luciferase assays demonstrated interaction between TEAD1 and BET protein BRD4, leading to the binding and activation of the Wnt4 promoter. In conclusion, TEAD1 is an essential regulator of the pro-fibrotic CFs phenotype associated with pathological cardiac remodeling via the BRD4/Wnt4 signalling pathway.

## Introduction

Heart failure (HF) remains a major contributor to global morbidity and mortality, with its incidence and prevalence steadily on the rise.^1^ Despite advances in prevention and treatment, the outcomes of HF often remain unsatisfactory. The process of cardiac remodeling involves intricate alterations in both the structure and function of the heart, encompassing both physiological adaptations and pathological changes. Physiological remodeling refers to the normal, adaptive responses of the heart to various conditions, such as regular exercise, pregnancy, or growth during childhood. In contrast, pathological cardiac remodeling refers to maladaptive changes that occur in response to various diseases or conditions, such as hypertension, HF, or myocardial infarction.^2^ This process is hallmarked by cardiomyocytes (CMs) hypertrophy, cardiac fibroblasts (CFs) activation, and extracellular matrix (ECM) deposition occurring as a reaction to various cardiac stress. From these pathological processes, phenotypic changes in CFs can exacerbate extracellular matrix (ECM) deposition and cardiac fibrosis, eventually leading to decreased compliance of the cardiac tissue and the eventual onset of advanced-stage HF. However, which critical factors driving CFs differentiation and cardiac remodeling in HF and the molecular mechanisms at the cellular level remain unclear.

Under normal conditions, mature fibroblasts are scattered within the myocardium, maintaining the ECM. Consequently, their proliferation and the expression of smooth muscle α‑actin (α‑SMA) or periostin remain at minimal levels. However, certain stimuli, such as pressure overload and neurohumoral stimulation, can trigger CFs to transdifferentiate into highly proliferative and migratory myofibroblasts, which express α‑SMA and have contractile capacities.^3^ Previous studies have demonstrated the predominant involvement of fibrogenic growth factors like transforming growth factor-β (TGF-β) and neurohormonal factors such as angiotensin II (Ang II) in the majority of cardiac fibrotic conditions. Context-specific control of gene expression predominantly occurs through the action of transcription factors (TFs). In response to mechanical stress, the gene expression of activated CFs is largely regulated by TFs.^4,5^ Cardiac TFs have been reported to intricately regulate the expression of genes associated with cardiac remodeling, such as GATA4, ETV1, and ETS2.^6–8^ Given the role of TFs in coordinating varied gene expressions in the mature heart when exposed to neurohormonal triggers, they stand as promising targets for therapeutic interventions. One potential approach involves developing drug compounds or decoy oligodeoxynucleotides that can specifically inhibit the activity of prohypertrophic TFs, thus holding therapeutic promise. Additionally, enhancing the functions of antihypertrophic TFs presents another avenue. Crucially, identifying endogenous TFs that promote cardiac remodeling may be crucial for designing novel therapeutics to treat pathological cardiac remodeling and HF.

Using a discovery-driven, unbiased approach, we have identified TEA domain transcription factor 1 (TEAD1) as a TF enriched in CFs and upregulated in pathological cardiac remodeling. TEAD1, a greatly conservative protein, is part of the TEA domain transcription factor (TEAD) family consisting of four transcription factors (TEAD1–4). Within this family, TEAD1, the most prevalent in the heart, acts as a pioneering factor. It exerts regulatory functions by binding to the canonical cytidine-adenosine-thymidine (MCAT) element and engages with diverse cofactors like yes-associated protein (YAP) and transcriptional coactivator with PDZ-binding motif (TAZ). Previous studies suggested that global deletion of TEAD1 or cardiomyocytes (CMs)- specific TEAD1 deficiency led to embryonic lethality or perinatal death due to a decrease in CM proliferation.^9,10^ Furthermore, eliminating TEAD1 in adult mouse cardiomyocytes leads to the rapid onset of lethal dilated cardiomyopathy, marked by significant impairment in excitation-contraction coupling^11^ and mitochondrial defects.^12,13^ Since above results highlight the unique function of TEAD1 in controlling fundamental cardiomyocyte-specific functions, it is thus resonable to suggest that TEAD1 might have a crucial role in the transcriptional regulation of cardiac fibroblast differentiation, a process critical for cardiac remodeling. Previous studies showed that TEAD1 could regulate genes associated with both heart development and cardiac remodeling, including the involvement of TEAD activity in CFs activation and in cardiomyocyte-elicited pro-fibrotic signals to cardiac fibroblasts that promote myocardial fibrosis.^14–16^ However, the exact regulation mechanisms are still uncovered. Therefore, the role of TEAD1 in CFs during cardiac remodeling was explored in this study.

In this study, we aimed to explore the involvement of TEAD1 in the cardiac remodeling induced by transverse aortic constriction (TAC) and angiotensin II (Ang-II). We observed that TEAD1 levels were significantly increased in humans and mice remodeling heart. By generating fibroblasts and myofibroblasts-specific TEAD1 knockout mice, we found that TEAD1 deficiency attenuated overload and Ang-II-induced cardiac remodeling and prevented cardiac dysfunction. Furthermore, TEAD1 knockdown effectively attenuated Ang II-induced CFs activation in vitro, whereas TEAD1 overexpression enhanced these changes. Transcriptomic and functional analyses revealed that Wnt4 is a novel TEAD1 target that contributes to cardiac remodelling by activating the Wnt signalling pathway, and genetic Wnt4 knockdown rescued the pro-transformation phenotype in TEAD1 overexpression fibroblasts. Coimmunoprecipitation demonstrated that TEAD1 regulated Wnt4 expression by interacting with the bromodomain protein BRD4.These findings demonstrate that TEAD1 may establish a foundation for future investigations of its therapeutic potential for cardiac remodelling and HF.

## Results

### TEAD1 expression is elevated in response to pathological cardiac remodeling in humans and mice

**Fig. 1:**
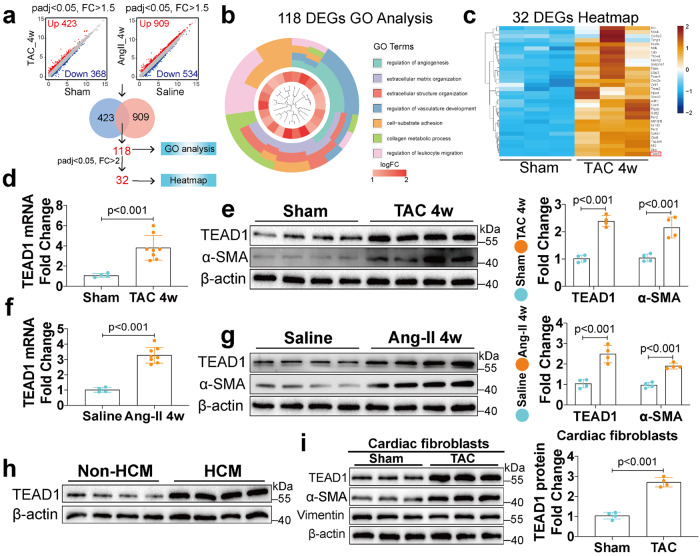
TEAD1 expression is increased in human and mouse remodeling hearts. **a** Schematic illustration of the RNA-seq analysis strategy. the blue dots represent DEGs with foldchange < 1/1.5, adjust *P* value < 0.05, the red dots represent DEGs with foldchange >1.5, adjust *P* value < 0.05. Grey dots represent gene that the expression change had no significant difference. The number of genes upregulated in TAC and Ang-II samples were indicated in the Venn diagram. **b** GO term enrichment analysis of 118 genes upregulated in both TAC and Ang-II samples (foldchange >1.5, adjust *P* value < 0.05). **c** Heatmap of 32 genes upregulated in both TAC and Ang-II samples (foldchange >2, adjust *P* value < 0.05). **d** Quantitative real time polymerase chain reaction (qRT-PCR) analyses of TEAD1 mRNA levels in heart samples from WT mice at 4 weeks after Sham or TAC (*n* = 4 for Sham and *n* = 8 for TAC per group). **e** Western blot and quantification of TEAD1 and α‑SMA protein levels in heart samples from WT mice at 4 weeks after Sham or TAC (*n* = 4 per group). **f** qRT-PCR analyses of TEAD1 mRNA levels in heart samples from WT mice at 4 weeks after Saline or Ang-II infusion (*n* = 4 for Saline and *n* = 8 for Ang-II per group). **g** Western blot and quantification of TEAD1 and α‑SMA protein levels in heart samples from WT mice at 4 weeks after Saline or Ang-II infusion (*n* = 4 per group). **h** Western blot of TEAD1 in heart samples from non-HCM and HCM patients. For all statistical plots, the data are presented as mean ± SD **i** Western blot and quantification of TEAD1, α‑SMA and Vimentin protein levels in isolated CFs at 4 weeks after TAC or Sham (*n* = 4 per group). **d-g** and **i** by two-tailed unpaired Student’s *t*-test. TAC, transverse aortic constriction; α-SMA, α-smooth muscle actin

To identify potential key regulators associated with cardiac remodeling, we performed RNA-sequencing (RNA-seq) analyses in myocardial samples from mice subjected to TAC or Ang-II infusion for 4 weeks (Fig. 1a). Overall, 423 and 909 genes were upregulated (false discovery rate q-value < 0.05; fold change > 1.5) in the TAC samples and Ang-II samples compared with the control samples, respectively (Fig. 1a). Of these, 118 genes were simultaneously upregulated in the TAC and Ang-II models (Fig. 1a). Gene Ontology (GO) analysis (false discovery rate q-value < 0.05) of 118 genes suggested that they were involved in ECM organisation, angiogenesis, and other cardiac remodeling process (Fig. 1b). Further analysis with fold change > 2 undergoing hierarchical clustering revealed 32 genes with elevated expression in both TAC and Ang-II models, of which we found that TEAD1 was particularly significantly elevated (Fig. 1c). To confirm the RNA-seq results, TEAD1 expression was further assessed in wild-type (WT) mice after TAC surgery or Ang-II infusion. Consistently, myocardial TEAD1 mRNA and protein levels increased 4 weeks post TAC and Ang-II infusion (Fig. 1d–g). Next, we determined whether these changes in TEAD1 expression also occurred in hypertrophic cardiomyopathy (HCM) patients. As expected, the expression of TEAD1 was upregulated in the HCM (Fig. 1h and Supplementary Fig. 1a). Altogether, these results suggest that the transcription factor TEAD1 may be associated with the development of pathological cardiac remodeling.

Next, we examined the potential cardiac cell populations responsible for elevated TEAD1 expression in response to cardiac remodeling. CMs and CFs were isolated from the hearts of adult mice subjected to TAC, and TEAD1 expression was evaluated using quantitative reverse transcription polymerase chain reaction (qRT-PCR) and western blot. Low TEAD1 expression was detected in both cardiac cell populations from the sham-operated mice. There was a marked elevation observed in both the mRNA and protein expression of TEAD1 within CFs after TAC (Fig. 1i). In contrast, TAC had no significant effect on the protein levels of TEAD1 in CMs (Supplementary Fig. 1b). Immunofluorescence staining also showed that TEAD1 was upregulated in tissue sections after TAC (Supplementary Fig. 1c). These results revealed that CFs-derived TEAD1 may play a key role in cardiac remodeling.

### TEAD1 knockout in CFs ameliorates TAC- and Ang-II-induced cardiac remodeling and dysfunction

**Fig. 2:**
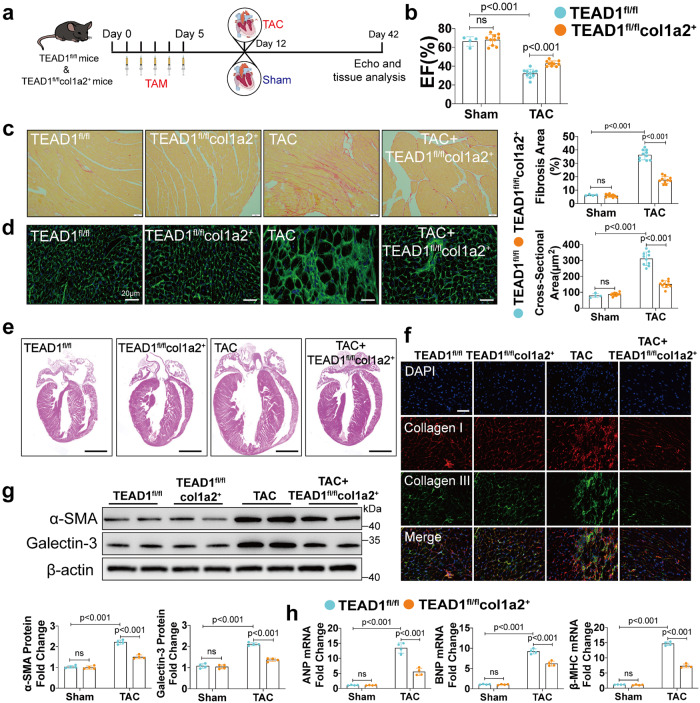
Knockout of TEAD1 in cardiac fibroblasts attenuates TAC-induced cardiac remodeling. **a** Schematic for echocardiography and sample collection from 4 groups: TEAD1^fl/fl^ and TEAD1^fl/fl^col1a2^+^ at 4 weeks after sham or TAC. **b** Left ventricular EF assessed by echocardiography in TEAD1^fl/fl^ and TEAD1^fl/fl^col1a2^+^ mice after 4 weeks sham or TAC (*n* = 4–10 per group). **c** Heart sections from TEAD1^fl/fl^ and TEAD1^fl/fl^col1a2^+^ mice after sham or TAC surgery were stained with picrosirius red to visualize collagen deposition (*n* = 4–10 mice per group; scale bar=50 μm). **d**. Heart sections from TEAD1^fl/fl^ and TEAD1^fl/fl^col1a2^+^ mice after sham or TAC surgery were stained with WGA to demarcate the cell boundaries (*n* = 4-10 mice per group; scale bar=20 μm). **e** Heart sections from TEAD1^fl/fl^ and TEAD1^fl/fl^col1a2+ mice after sham or TAC surgery were stained with hematoxylin and eosin to show whole-heart gross images (*n* = 4–10 mice per group; scale bar=5 mm). **f** Representative immunofluorescence images of Collagen I and Collagen III staining in the hearts from TEAD1^fl/fl^ and TEAD1^fl/fl^col1a2^+^ mice after sham or TAC surgery (*n* = 4 per group; scale bar=20 μm). **g** Western blot and quantification of α‑SMA and Galectin-3 protein levels in the heart homogenates extracted from TEAD1^fl/fl^ and TEAD1^fl/fl^col1a2^+^ mice after sham or TAC surgery (*n* = 4 per group). **h** qRT-PCR analyses of the mRNA levels of ANP, BNP and β-MHC in heart samples from TEAD1^fl/fl^ and TEAD1^fl/fl^col1a2^+^ mice after sham or TAC surgery (*n* = 4 per group). For all statistical plots, the data are presented as mean ± SD. ns. indicates no significance between the 2 indicated groups. **b**–**d** and **g** by two-way ANOVA with Bonferroni multiple comparison test. h by two-way ANOVA with Dunnett’s T3 post hoc analysis. TAM, tamoxifen; EF, ejection fraction; WGA, wheat germ agglutinin; ANP, atrial natriuretic peptide; BNP, brain natriuretic peptide; β-MHC, β-myosin heavy chain

Next, we explored the in vivo effects of TEAD1 knockout in mice. Knockout of TEAD1 in CFs (TEAD1^fl/fl^col1a2^+^) was generated by crossing TEAD1-floxed (TEAD1^fl/fl^) mice with col1a2-cre/ERT mice, thus allowing tamoxifen (TAM)-inducible deletion of TEAD1 in CFs (Supplementary Fig. 2a). The genotype of TEAD1^fl/fl^ and col1a2^+^ mice was determined through PCR analysis (Supplementary Fig. 2b). TAM administation for 5 days successfully reduced the expression of TEAD1 in CFs from TEAD1^fl/fl^col1a2^+^ mice, while TEAD1 expression remained normal in CFs from the control TEAD1^fl/fl^ mice not expressing Cre recombinase (Supplementary Fig. 2c, d). This decrease was specific to CFs (Supplementary Fig. 2e, f). After continuous administration of TAM for 5 days, TEAD1^fl/fl^col1a2^+^ and TEAD1^fl/fl^ mice had 7 days to acclimate the effects of TAM and then were performed TAC for 4 weeks (Fig. 2a). Although no remarkable changes were observed at the basal level, the TEAD1^fl/fl^col1a2^+^ mice exhibited significantly improved ejection fraction (EF) values after TAC compared with the TEAD1^fl/fl^ mice (Fig. 2b). Specific cardiac ultrasound parameters were shown in Supplementary Table 3. Sirius red and immunofluorescence staining showed that TAC-induced fibrosis was significantly attenuated in the TEAD1^fl/fl^col1a2^+^ mice compared with the TEAD1^fl/fl^ mice (Fig. 2c, f). Wheat germ agglutinin (WGA) staining revealed a significantly reduced CMs cross-sectional area (CSA) in the TEAD1^fl/fl^col1a2^+^ mice compared with that in the TEAD1^fl/fl^ mice (Fig. 2d). Whole-heart gross images also showed that TEAD1 knockout in CFs reduced hypertrophy induced by TAC (Fig. 2e). Further investigation revealed the reduced levels of cardiac fibrotic markers α‑SMA and galectin-3 in the TEAD1^fl/fl^col1a2^+^ mice after TAC compared with the TEAD1^fl/fl^ mice (Fig. 2g). The atrial natriuretic peptide (ANP), brain natriuretic peptide (BNP), and beta-myosin heavy chain (β-MHC) expression was also dramatically inhibited in the TEAD1^fl/fl^col1a2^+^ mice (Fig. 2h).

We next investigated whether TEAD1 deficiency could elicit similar cardioprotective effects in response to neurohumoral cardiac stress. After continuous administration of TAM for 5 days, TEAD1^fl/fl^col1a2^+^ and TEAD1^fl/fl^ mice had 7 days to acclimate the effects of TAM and then were infused with Ang-II for 4 weeks via an implantable minipump (Supplementary Fig. 3a). Echocardiography revealed no significant differences between the EF values of the TEAD1^fl/fl^col1a2^+^ and TEAD1^fl/fl^ mice following Ang-II infusion (Supplementary Fig. 3b). Specific cardiac ultrasound parameters were shown in Supplementary Table 4. Furthermore, consistent with what was observed in response to TAC, the TEAD1^fl/fl^col1a2^+^ mice exhibited a lower cardiac fibrosis and CSA after Ang-II infusion (Supplementary Fig. 3c, d). Additionally, the Ang-II-induced increase in cardiac fibrotic markers α‑SMA and galectin-3 was also significantly attenuated in the TEAD1^fl/fl^col1a2^+^ mice (Supplementary Fig. 3e). The ANP, BNP and β-MHC expression was also dramatically inhibited in the TEAD1^fl/fl^col1a2^+^ mice (Supplementary Fig. 3f). These results demonstrated that TEAD1 deficiency in CFs effectively prevented cardiac remodeling in response to TAC and Ang-II infusion.

### Myofibroblasts-specific TEAD1 deficiency attenuates stress-induced cardiac remodeling and dysfunction

**Fig. 3:**
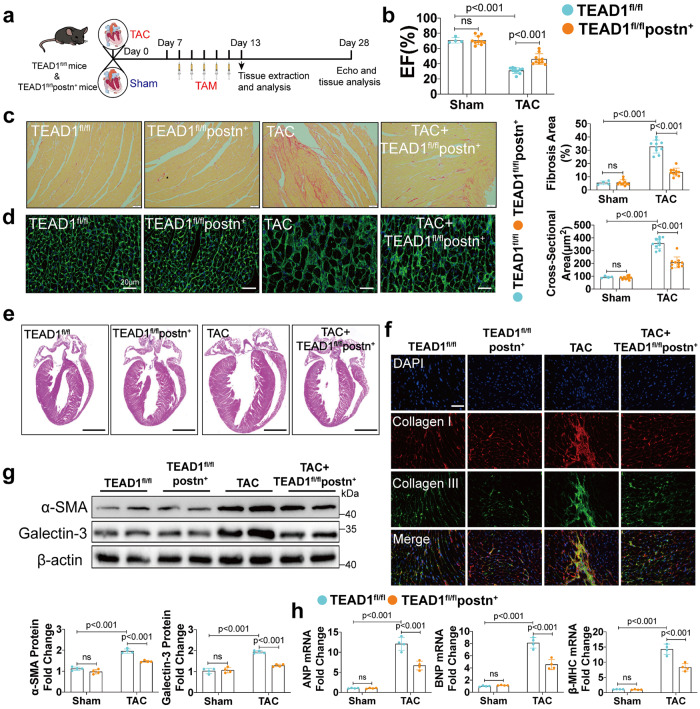
Myofibroblast-specific TEAD1 deficiency attenuates TAC-induced cardiac remodeling. **a** Schematic for echocardiography and sample collection from 4 groups: TEAD1^fl/fl^ and TEAD1^fl/fl^postn^+^ 4 weeks after 4 weeks sham or TAC. **b** Left ventricular EF assessed by echocardiography of TEAD1^fl/fl^ and TEAD1^fl/fl^postn^+^ mice after 4 weeks sham or TAC (*n* = 4-10 per group). **c** Heart sections from TEAD1^fl/fl^ and TEAD1^fl/fl^postn^+^ mice after sham or TAC surgery were stained with picrosirius red to visualize collagen deposition (*n* = 4-10 mice per group; scale bar=50 μm). **d** Heart sections from TEAD1^fl/fl^ and TEAD1^fl/fl^postn^+^ mice after sham or TAC surgery were stained with WGA to demarcate the cell boundaries (*n* = 4-10 mice per group; scale bar=20 μm). **e** Heart sections from TEAD1^fl/fl^ and TEAD1^fl/fl^postn+ mice after sham or TAC surgery were stained with hematoxylin and eosin to show whole-heart gross images (*n* = 4-10 mice per group; scale bar=5 mm). **f** Representative immunofluorescence images of Collagen I and Collagen III staining in the hearts from TEAD1^fl/fl^ and TEAD1^fl/fl^postn^+^ mice after sham or TAC surgery (*n* = 4 per group; scale bar=20 μm). **g** Western blot and quantification of α‑SMA and Galectin3 protein levels in the heart homogenates extracted from TEAD1^fl/fl^ and TEAD1^fl/fl^postn^+^ mice after sham or TAC surgery (*n* = 4 per group). **h**. qRT-PCR analyses of the mRNA levels of ANP, BNP and β-MHC in heart samples from TEAD1^fl/fl^ and TEAD1^fl/fl^postn^+^ mice after sham or TAC surgery (*n* = 4 per group). For all statistical plots, the data are presented as mean ± SD. ns. indicates no significance between the 2 indicated groups. **b**–**d** and **g** by two-way ANOVA with Bonferroni multiple comparison test. **h** by two-way ANOVA with Dunnett’s T3 post hoc analysis. TAM tamoxifen; EF ejection fraction; WGA wheat germ agglutinin; ANP atrial natriuretic peptide; BNP brain natriuretic peptide; β-MHC β-myosin heavy chain

To further explore the effects of TEAD1 deficiency in response to TAC, TEAD1^fl/fl^ mice were crossed with postn-MerCreMer mice to knockout TEAD1 in myofibroblast (TEAD1^fl/fl^postn^+^) (Supplementary Fig. 4a). PCR analysis was used to determine the genotyping of TEAD1^fl/fl^ and postn^+^ mice (Supplementary Fig. 4b). Postn has been characterized as a marker only responsive to injury in myofibroblasts from adult tissues.^17^ TEAD1^fl/fl^postn^+^ mice were subjected to TAC for 7 days to transform fibroblasts into myofibroblasts, TAM was then administrated at the 8^th^ day post TAC for subsequent 5 days (Fig. 3a). Knockout specificity in heart and myofibroblasts was verified at 13^th^ day after TAC by qRT-PCR and immunoblot analyses (Supplementary Fig. 4c–f). The TEAD1^fl/fl^postn^+^ mice exhibited significantly preserved EF values after TAC compared with the TEAD1^fl/fl^ mice (Fig. 3b). Specific cardiac ultrasound parameters were shown in Supplementary Table 5. Sirius red and immunofluorescence staining showed that TAC-induced fibrosis was significantly attenuated in the TEAD1^fl/fl^postn^+^ mice after TAC compared with the TEAD1^fl/fl^ mice (Fig. 3c, f). WGA staining revealed a significantly reduced CMs CSA in the TEAD1^fl/fl^postn^+^ mice compared with the TEAD1^fl/fl^ mice (Fig. 3d). Likewise, whole-heart gross images also showed that TEAD1 knockout in myofibroblasts reduced hypertrophy induced by TAC (Fig. 3e). The TAC-induced upregulation of cardiac fibrotic markers α‑SMA and galectin-3 was also attenuated in the TEAD1^fl/fl^postn^+^ mice (Fig. 3g). A significant reduction of ANP, BNP, and β-MHC was also observed in the TEAD1^fl/fl^postn^+^ mice after pressure overload (Fig. 3h).

We further confirmed the role of TEAD1 in myofibroblast in Ang-II-induced cardiac remodeling model. This conditional knockout of TEAD1 was induced in mice subjected to Ang-II for 7 days, then followed by administration of TAM for subsequent 5 days (Supplementary Fig. 5a). Knockout specificity in heart and myofibroblasts was verified at 13^th^ day after Ang-II by qRT-PCR and immunoblot analyses (Supplementary Fig. 4g–j). The mitigation of remodeling was similar with our previous TAC model. The difference in the EF was not observed between the TEAD1^fl/fl^postn^+^ and TEAD1^fl/fl^ mice post Ang-II treatment (Supplementary Fig. 5b). Specific cardiac ultrasound parameters were shown in Supplementary Table 6. Cardiac fibrosis was significantly reduced in the TEAD1^fl/fl^postn^+^ mice compared with the TEAD1^fl/fl^ mice after Ang-II stimulation (Supplementary Fig. 5c). The TEAD1^fl/fl^postn^+^ mice exhibited a significantly reduced CMs CSA after Ang-II stimulation (Supplementary Fig. 5d). The fibrotic markers α‑SMA and galectin-3 (Supplementary Fig. 5e) as well as ANP, BNP, and β-MHC (Supplementary Fig. 5f) were significantly reduced in the TEAD1^fl/fl^postn^+^ mice compared with the TEAD1^fl/fl^ mice after Ang-II stimulation. These results suggested that TEAD1 knockout in myofibroblasts may be an effective strategy to reverse cardiac remodeling after cardiac injury.

### TEAD1 inhibition and knockdown ameliorate CFs differentiation and collagen secretion in vitro

Our results prompted us to explore the role of TEAD1 in the pathological differentiation of CFs in vitro. We assessed whether the pharmacological inhibition of TEAD1 signalling via VT103, which is known to strongly inhibit the TEAD/Yes-associated protein (YAP) interaction, could recapitulate the beneficial effects of TEAD1 deficiency in CFs after stimulation with Ang-II. VT103 treatment did not affect the TEAD1 mRNA or protein expression levels in these cells (Supplementary Fig. 6a, b). However, as expected, co-immunoprecipitation assay revealed that VT103 treatment significantly inhibited the TEAD1/YAP interaction in vitro and in vivo (Supplementary Fig. 6c, d). RNA-seq analysis with VT103 revealed that 41 genes were upregulated in the NMCFs (Neonatal Mice Cardiac Fibroblasts) after Ang-II stimulation, but this upregulation was reversed by VT103 treatment (Supplementary Fig. 7a). Gene Ontology (GO) analysis (false discovery rate q-value < 0.05) revealed that genes downregulated in response to VT103 treatment were associated with ECM organisation and cell adhesion (Supplementary Fig. 7b). Kyoto Encyclopaedia of Genes and Genomes analysis (KEGG, false discovery rate q-value < 0.05) further revealed the Wnt signalling pathway as the most significantly enriched category (Supplementary Fig. 7c). Immunofluorescence staining confirmed that Ang-II stimulation significantly increased α-SMA expression and that this effect was reversed by VT103 treatment (Supplementary Fig. 7d). Next, Ang-II increased collagen gel contraction over a 48-hour period, but this effect was inhibited by VT103 treatment (Supplementary Fig. 7e). VT103 also ameliorated Ang-II-induced fibroblasts migration (Supplementary Fig. 7f). Ang-II-induced increased expression of genes associated with extracellular matrix deposition was abolished with VT103 treatment in NMCFs (Supplementary Fig. 7g).

**Fig. 4:**
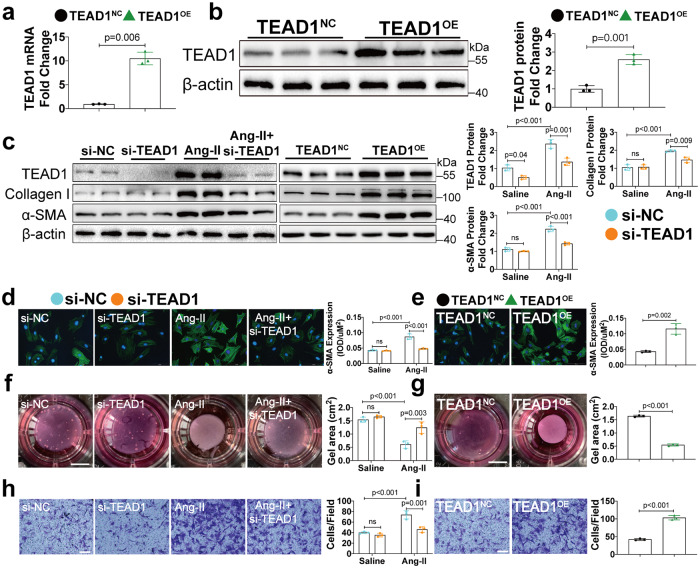
TEAD1 regulates Ang-II-induced fibroblast-to-myofibroblast transition in vitro. **a** qRT-PCR analyses of the mRNA levels of TEAD1 in CFs infected with adenovirus expressing TEAD1 or NC (*n* = 3 per group). **b** Western blot and quantification of TEAD1 protein levels in CFs infected with indicated adenovirus (*n* = 3 per group). **c**. Western blot and quantification of TEAD1, Collagen I and α‑SMA protein levels in CFs transfected with si-NC or si-TEAD1 and then treated with saline or Ang-II for 48 h (*n* = 3 per group) or infected with indicated adenovirus (*n* = 3 per group). **d** Representative images of immunofluorescence staining and quantification of α‑SMA in CFs transfected with si-NC or si-TEAD1 and then treated with saline or Ang-II for 48 h (*n* = 3 per group, scale bar=100 μm). **e**. Representative images of immunofluorescence staining against α-SMA and quantification of the CFs infected with indicated adenovirus (*n* = 3 per group, scale bar=100 μm). **f** Collagen gel contraction seeded CFs transfected with si-NC or si-TEAD1 and then treated with saline or Ang-II for 48 h (*n* = 3 per group). **g** Collagen gel contraction seeded CFs infected with indicated adenovirus (*n* = 3 per group). **h**. Migration of CFs transfected with si-NC or si-TEAD1 and then treated with saline or Ang-II (*n* = 3 per group; scale bar=100 μm). **i**. Migration of CFs infected with indicated adenovirus (*n* = 3 per group; scale bar=100 μm). For all statistical plots, the data are presented as mean ± SD. ns. indicates no significance between the 2 indicated groups. a by Welch’s *t*-test. **b, c, e, g,** and **i** by two-tailed unpaired Student’s *t*-test; **c, d, f** and **h** by two-way ANOVA with Bonferroni multiple comparison test

Since VT103 only occupies the interface on TEAD1 and disrupts YAP-TEAD1 interaction in cells^18,19^ and thus cannot reduce the TEAD1 protein expression, we next determined whether specific knockdown of TEAD1 by siRNA would phenocopy the effects of VT103 on suppressing the CFs differentiation and whether adenovirus expressing TEAD1 would promote the activation of CFs. After adenovirus infection, significantly elevated TEAD1 mRNA and protein levels were confirmed in NMCFs (Fig. 4a, b). Transfection with siRNA successfully knocked down TEAD1 protein levels in NMCFs (Fig. 4c). Consistent with our findings in antagonist treatment, the increases of TEAD1, α-SMA and collagen I at protein level were reversed by TEAD1 knockdown in NMCFs (Fig. 4c). However, overexpression of TEAD1 alone can induce upregulation of α-SMA and collagen I protein levels in NMCFs (Fig. 4c). Moreover, TEAD1 knockdown significantly reduced α-SMA expression, collagen gel contraction, and cell migration following Ang-II stimulation (Fig. 4d, f and h), whereas TEAD1 overexpression enhanced these effects (Fig. 4e, g and i). These findings further supported an association between TEAD1 expression and Ang-II-induced CFs differentiation and that these effects could be effectively attenuated via TEAD1 knockdown.

### TEAD1 regulates CFs differentiation via the bromodomain-containing protein 4 (BRD4)/Wnt4 signalling pathway

**Fig. 5:**
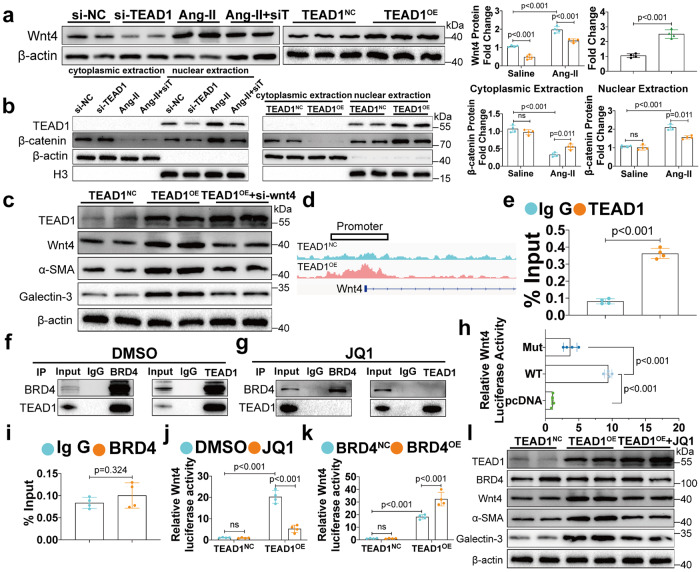
TEAD1 regulates CFs differentiation via the bromodomain-containing protein 4 (BRD4)/Wnt4 signalling pathway. **a** Western blot and quantification of Wnt4 protein levels in CFs transfected with si-NC or si-TEAD1 and then treated with saline or Ang-II for 48 h (*n* = 4 per group); Western blot and quantification of Wnt4 protein levels in CFs infected with adenovirus expressing TEAD1 or NC (*n* = 4 per group). **b** Western blot and quantification of TEAD1 and β-catenin protein levels in cytoplasmic and nuclear fractions extracted from CFs transfected with si-NC or si-TEAD1 and then treated with saline or Ang-II (*n* = 4 per group); Western blot and quantification of TEAD1 and β-catenin protein levels in cytoplasmic and nuclear fractions extracted from CFs infected with adenovirus expressing TEAD1 or NC (*n* = 4 per group). **c** Western blot of TEAD1, Wnt4, α‑SMA and Galectin-3 protein levels in CFs infected with adenovirus expressing TEAD1 and transfected with si-Wnt4. **d** TEAD1 binding tracks at Wnt4 gene loci in CFs based on CHIP-seq dataset. **e** ChIP-qPCR analysis using a TEAD1-specific antibody to detect TEAD1 binding to the Wnt4 promoter in CFs (*n* = 4 per group). **f**–**g** Endogenous immunoprecipitation of TEAD1 and BRD4 in the presence (**f**) or absence (**g**) of JQ1. **h** Luciferase activity in HEK293T transfected with WT or mutating Wnt4 luciferase reporter plasmids (*n* = 4 per group). **i**. ChIP-qPCR analysis using an BRD4-specific antibody to detect BRD4 binding to the Wnt4 promoter in CFs (*n* = 4 per group). **j**. Luciferase activity in CFs transfected with a Wnt4 luciferase reporter plasmid, along with adenovirus expressing TEAD1 in the presence or absence of JQ1 (1 μM) (*n* = 4 per group). **k** Luciferase activity in CFs transfected with an Wnt4 luciferase reporter plasmid, along with adenovirus expressing indicated proteins (*n* = 4 per group). **l** Western blot of TEAD1, BRD4, Wnt4, α‑SMA and Galectin-3 protein levels in CFs infected with adenovirus expressing TEAD1 in the presence or absence of JQ1. For all statistical plots, the data are presented as mean ± SD. ns. indicates no significance between the 2 indicated groups. **a**–**b** by two-way ANOVA with Bonferroni multiple comparison test. **e** and **i** by two-tailed unpaired Student’s *t*-test. **j**–**k** by two-way ANOVA with Dunnett’s T3 multiple comparisons test. **h** by one-way ANOVA with Dunnett’s T3 multiple comparisons test. ChIP, chromatin immunoprecipitation

To fully understand the molecular mechanisms underlying TEAD1-mediated cardiac remodeling, we next performed genome-wide RNA-seq to identify the transcriptomic changes in response to TEAD1 knockdown and overexpression in NMCFs. Hierarchical clustering analysis of all identified genes revealed six clearly defined clusters that were associated with their respective experimental cohorts (si-NC, si-TEAD1, Ang-II, si-TEAD1+Ang-II, OE-NC, and OE-TEAD1); hence we performed additional differential gene expression analysis (Supplementary Fig. 8a). Overall, 77 genes were upregulated in the NMCFs following Ang-II stimulation, but this upregulation was reversed by si-TEAD1 (Supplementary Fig. 8c). Furthermore, 20 of these 77 differentially expressed genes were upregulated after TEAD1 overexpression (Supplementary Fig. 8b). GO analysis (false discovery rate q-value < 0.05) revealed genes that were downregulated in response to TEAD1 knockdown were associated with ECM organisation, extracellular structure organisation, and angiogenesis, all of which are known to be involved in cardiac remodeling (Supplementary Fig. 8d). KEGG analysis (false discovery rate q-value < 0.05) further revealed the Wnt signalling pathway as the most significantly enriched category (Supplementary Fig. 8e). Notably, gene set enrichment analysis (GSEA) (false discovery rate q-value < 0.05) revealed that the Wnt signalling pathway was significantly downregulated in response to TEAD1 knockdown and significantly upregulated in response to TEAD1 overexpression (Supplementary Fig. 8f, g). Among the differential genes regulating the Wnt signaling pathway, we found that Wnt4 was significantly down-regulated in si-TEAD1 and up-regulated in TEAD1-OE (Fig. 5a). Interestingly, the Wnt4 expression was also upregulated in the HCM patients (Supplementary Fig. 9a, b). We found that under no matter pathological stimulation or TEAD1 overexpression, TEAD1 was almost absent in the cytoplasm (Fig. 5b). Furthermore, we found that si-TEAD1 alone did not significantly affect the nuclear translocation of β-catenin but prevented beta-catenin nuclear import in the presence of Ang-II stimulation, suggesting that si-TEAD1 might sensitively function in response to neurohumoral stimulation instead of under physiological homeostasis (Fig. 5b). Interestingly, overexpression of TEAD1 alone could induce the nuclear import of β-catenin (Fig. 5b), which was connected with the previous results that overexpression of TEAD1 alone could induce the differentiation of CFs. There were no notable alterations detected in the protein levels of c-Jun N-terminal kinase (JNK) or calcium/calmodulin-dependent protein kinase II (CaMKII), indicating that the non-canonical Wnt signalling pathway remained inactivated (Supplementary Fig. 10a, b). In vivo, decreased Wnt4 expression was observed in mice with knocking out TEAD1 in CFs or myofibroblasts following TAC or Ang-II infusion (Supplementary Fig. 11a–d).

Next, Wnt4 was knocked down in NMCFs using siRNA to confirm the requirement of Wnt signalling in TEAD1-induced CFs differentiation. Indeed, Wnt4 deficiency reduced the expression of α-SMA and Galectin-3 induced by TEAD1 overexpression (Fig. 5c). Furthermore, we used existing CFs-specific TEAD1 knockout mice and injected AAV9-Tcf21-Wnt4 to observe whether Wnt4 overexpression in CFs would block the protection provided by TEAD1 knockout after TAC (Supplementary Fig. 12a). Wnt4 expression was significantly upregulated in CFs after AAV9 injection (Supplementary Fig. 12b). Wnt4 overexpression blocked the protective effect of TEAD1 knockout after TAC, mainly reflected by EF, FS (Supplementary Fig. 12d, e), HE staining (Supplementary Fig. 12c), sirius red staining (Supplementary Fig. 12f) and fibrotic markers (Supplementary Fig. 12g). These results illustrated the importance of Wnt4 activity as an essential downstream signaling pathway in TEAD1-mediated CFs transformation. TEAD1^NC^ and TEAD1^OE^ were subjected to whole-genome chromatin immunoprecipitation-sequencing (ChIP-seq) and the gene regulatory elements bound by TEAD1 were mapped. TEAD1 was enriched at the promoters, with 15.56% of the bound regions located within 2 kb of transcription start site (TSS). Moreover, TEAD1 overexpression increased TEAD1 chromatin occupancy within the promoter of Wnt4 (Fig. 5d). Chromatin immunoprecipitation (ChIP)-qPCR confirmed high enrichment efficiency of the Wnt4 promoter with the TEAD1 antibody than anti-IgG antibody (Fig. 5e), indicating TEAD1 serves as a transcriptional regulator of Wnt4. The ChIP-seq predicted TEAD1 binding sites within the Wnt4 promoter located at -526-516. To further confirm the above binding site, the mutating plasmid within the TEAD1 binding sites in Wnt4 promoter was transfected into HEK293T cells. Significantly decreased Wnt4 promoter activity was detected in missing mutants compared with the WT fragment (Fig. 5h).

We found that knocking down YAP could reduce the expression of Wnt4 (Supplementary Fig. 13a), confirming that both TEAD1 and YAP could regulate the expression of Wnt4. Our research is focused on identifying additional proteins or co-factors that interact with TEAD1 and YAP to influence their transcriptional activity. To further investigate the mechanisms underlying TEAD1-induced CFs differentiation through Wnt signalling pathway, MS was performed to identify proteins that specifically interacted with TEAD1. Among the co-purified proteins, BRD4, an important regulator of CFs differentiation, was found to be enriched among the purified protein complexes. Co-immunoprecipitation assays were performed to confirm this direct interaction between TEAD1 and BRD4 in CFs (Fig. 5f). However, after treatment with VT103, TEAD1 cannot bind to YAP or BRD4 (Supplementary Fig. 13b, c). In addition, we found that VT103 did not affect the activation status of YAP induced by Ang-II (Supplementary Fig. 13d). We speculate that VT103 mainly affects the binding of TEAD1 to YAP or BRD4 in the nucleus, but has no effect on phosphorylated YAP in the cytoplasm. After VT103 treatment, TEAD1 was inactivated and could not bind to the promoter of Wnt4, thus losing the cooperation with BRD4. Additionally, blocking bromodomain (BD) of BRD4 through the inhibitor JQ1 affected the binding of BRD4 and TEAD1 (Fig. 5g). Next, we tested whether BRD4 directly regulates the expression of Wnt4 and thus affects the downstream Wnt signaling pathway and fibroblast transformation. ChIP-qPCR assay results indicated that there was no BRD4 binding site in the promoter region of Wnt4 (Fig. 5i). Furthermore, TEAD1-activated luciferase reporter activity driven by Wnt4 promoter was reduced in response to JQ1 treatment (Fig. 5j), while co-transfection of both TEAD1 and BRD4 resulted in additive activation of this reporter (Fig. 5k). Next, qRT-PCR and Western blot results showed that the BRD4 inhibitor JQ1 could indeed inhibit the increase of Wnt4 and α-SMA expression caused by TEAD1 overexpression (Supplementary Fig. 14a and Fig. 5l). Moreover, JQ1 treatment significantly reduced α-SMA expression, collagen gel contraction, and cell migration following TEAD1 overexpression (Supplementary Fig. 14d–f). More significant upregulation of Wnt4 and ACTA2 expression was also evidenced upon co-transfection of both TEAD1 and BRD4 compared with transfection of TEAD1 alone (Supplementary Fig. 14b, c). Taken together, TEAD1 and BRD4 interact in a bromodomain-dependent manner to control the differentiation of CFs, suggesting BRD4 can be preferentially targeted to specific genomic loci by combination with TEAD1.

### TEAD1 inhibitor VT103 exhibits promising pharmacological effects for preventing stress-induced cardiac remodeling

**Fig. 6:**
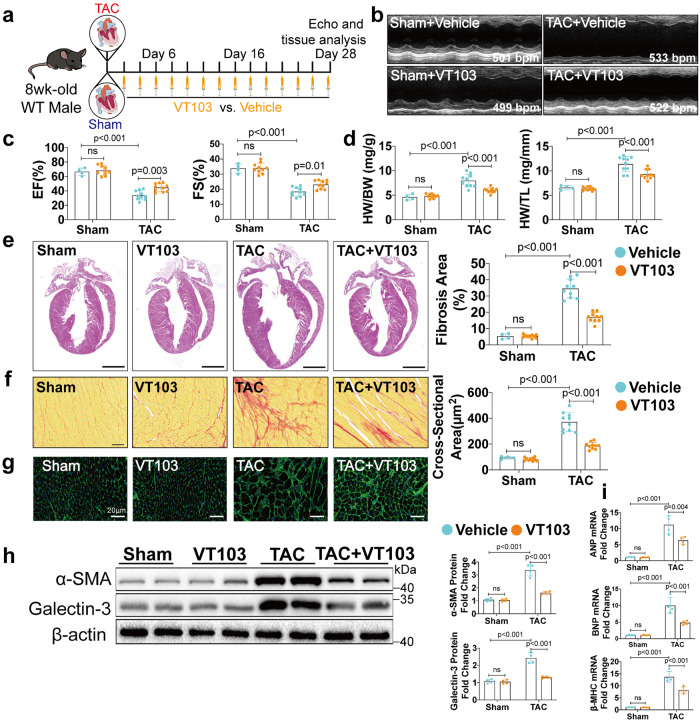
VT103 attenuates TAC-induced cardiac remodeling by inhibiting TEAD1. **a** Schematic for echocardiography and sample collection from 4 groups: WT mice were subjected to sham or TAC subsequently treated with vehicle or VT103 (20 mg/kg/day) every 2 days via intraperitoneal injection for 28 days (*n* = 4–10 per group). **b** Representative echo image of M-mode after 4 weeks TAC or sham. **c** Left ventricular EF and FS assessed by echocardiography in WT mice after 4 weeks sham or TAC subsequently treated with vehicle or VT103 (*n* = 4–10 per group). **d** The ratios of HW to BW and HW to TL in WT mice after 4 weeks sham or TAC subsequently treated with vehicle or VT103 (*n* = 4–10 per group). **e**–**g** Heart sections were stained with hematoxylin and eosin, WGA or picrosirius red from WT mice subjected to sham or TAC surgery subsequently treated with vehicle or VT103 (*n* = 4–10 per group; for hematoxylin and eosin staining, scale bar=5 mm; for WGA staining, scale bar=20 μm; for picrosirius red staining, scale bar = 50 μm). **h** Western blot and quantification of α‑SMA and Galectin-3 protein levels in the heart homogenates extracted from WT mice after sham or TAC surgery treated with vehicle or VT103 (*n* = 4 per group). **i** qRT-PCR analyses of the mRNA levels of ANP, BNP and β-MHC in heart homogenates extracted from WT mice after sham or TAC surgery treated with vehicle or VT103 (*n* = 4 per group). For all statistical plots, the data are presented as mean ± SD. ns. indicates no significance between the 2 indicated groups. **c**–**d** and **f**–**h** by two-way ANOVA with Bonferroni multiple comparison test. **i** by two-way ANOVA with Dunnett’s T3 post hoc analysis

To investigate the potential therapeutic possibility of TEAD1 inhibition in cardiac remodeling, we evaluated the effects of the TEAD1 inhibitor VT103 on TAC-induced cardiac remodeling. To test the in vivo effects of VT103, we intraperitoneally administered VT103 (20 mg/kg every other day for 28 days) to mice 3 days after TAC or sham surgery (Fig. 6a). The safety of TEAD1 inhibitors VT103 was evaluated during the implementation of the experiment. We found that the use of VT103 did not affect the body weight and heart rate as well as physiological functions of major organs (liver and kidney) in mice through histological (HE staining) and biochemical analyses (creatine kinase, LDH, AST, ALT and creatinine) (Supplementary Fig. 15a–g). VT103 treatment prevented TAC-induced cardiac hypertrophy and dysfunction in WT mice, as demonstrated by preserved EF and FS values and lower HW/BW and HW/TL ratios in these mice compared with the vehicle control (Fig. 6b–d). Specific cardiac ultrasound parameters were shown in Supplementary Table 7. Consistently, histological analyses revealed that VT103 reduced the cardiac hypertrophy, CMs CSA and fibrotic area following TAC in these mice (Fig. 6f, g). Additionally, VT103 treatment reduced the expression of cardiac fibrotic markers α‑SMA and galectin-3 as well as ANP, BNP, and β-MHC, which were all upregulated in the vehicle control (Fig. 6h, i). These data reinforced a predominant role of TEAD1 in cardiac remodeling and suggested the clinical translation of TEAD1 inhibitor in treating cardiac diseases associated with pathological remodeling.

**Fig. 7:**
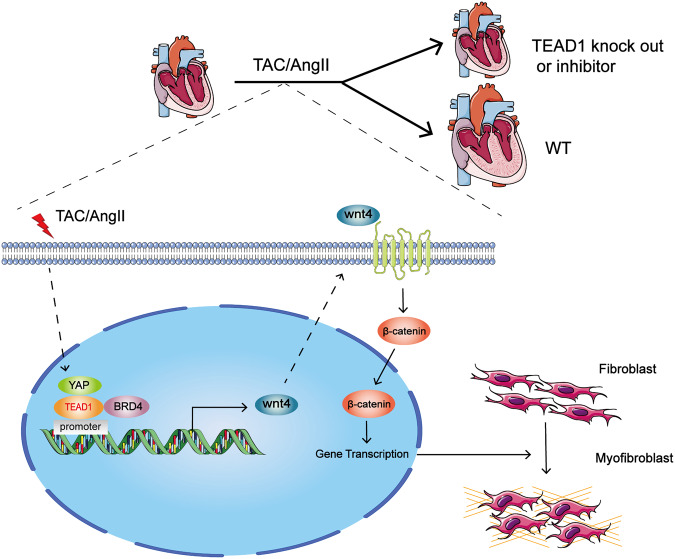
TEAD1 promotes the fibroblast-to-myofibroblast transition during TAC or Ang-II-induced pathological cardiac remodelling through the Wnt signalling pathway. TAC or Ang II stimulation induces TEAD1 expression, which binds to the promoter of Wnt4 to promote its expression through interaction with BRD4. The overexpressed Wnt4 enhances the nuclear translocation of β-catenin, thus activating the canonical Wnt signalling pathway to promote the fibroblast-to-myofibroblast transition. This figure was drawn by using pictures from Servier Medical Art (https://smart.servier.com/). Servier Medical Art by Servier is licensed under a Creative Commons Attribution 3.0 Unported License (https://creativecommons.org/licenses/by/3.0/)

## Discussion

In this study, we identified TEAD1 as a novel regulator of pathological cardiac remodeling and found that TEAD1 expression was upregulated in both human patients with cardiac remodeling as well as mice with chronic pressure overload induced by either TAC or Ang-II stimulation. We further demonstrated that this TEAD1 upregulation was selective to CFs but not CMs. Using CFs or myofibroblasts-TEAD1 knockout mice and TEAD1 inhibitor, we showed that TEAD1 deficiency ameliorated both TAC-and Ang-II-induced cardiac remodeling and TAC-induced cardiac dysfunction. Mechanically, TEAD1 interacted with BRD4 to activate the transcriptional activity of promoter Wnt4, thus promoting fibroblast transformation into the myofibroblast phenotype through the Wnt signalling pathway (Fig. 7). Taken together, this work has identified TEAD1 as a potential therapeutic target for inhibition of fibroblast-to-myofibroblast differentiation and prevention of the initiation and progression in cardiac remodeling and HF.

CFs are essential in maintaining the structural integrity of the heart and participate in various physiological and pathological processes, including cardiac remodeling.^20^ Under pathological conditions, various sources of CFs can differentiate into myofibroblasts. The origins of myofibroblasts have been debated for decades.^21^ Although it’s now generally accepted that they originate from existing fibroblasts through proliferation,^22^ Zhou et al. additionally recognized that fibroblasts originating from the endocardium played a critical role as a subset of myofibroblasts, contributing significantly to the development of severe cardiac fibrosis following pressure overload injury.^23^ The key characteristic of myofibroblasts, regardless of their source, contribute to the pathological remodeling of the heart tissue and the excessive deposition of collagen and other ECM components, ultimately leading to cardiac fibrosis. So unraveling the mechanisms of myofibroblast activation is crucial for developing targeted therapies to prevent or mitigate cardiac remodeling and cardiac dysfunction.

Both RNA-seq analysis from TAC and Ang-II infusion revealed the upregulation of TEAD1. Recently, it has been demonstrated that the TEAD1 may have different, or even opposite effects on global organ function depending on its expression in certain cell types.^24^ An increased expression of TEAD1 in the nucleus of CMs has been described in mice with cardiac-specific homozygous knockout of WW45 subjected to TAC, presented with cardiac remodeling.^25^ Relatedly, TEAD1 overexpression in CMs leads to an age-dependent dysfunction.^16^ It is undeniable that the expression of TEAD1 in CMs may play an important role in myocardial remodeling, but previous studies have demonstrated that cardiomyocyte-specific TEAD1 knockout mice resulted in an acute-onset dilated cardiomyopathy due to mitochondrial dysfunction.^11–13^ The inconsistent function of TEAD1 between knockout and overexpression imply that the role of CMs-elicited pro-fibrotic signals to CFs may involve in myocardial fibrosis.^26^ Our ex vivo findings demonstrated that CFs predominantly contributed to the TEAD1 upregulation in response to pressure overload. Therefore, it is necessary to systematically study the role and mechanism of TEAD1 in cardiac remodeling in CFs.

TEAD1/YAP plays critical roles in cardiac protection and regeneration.^27,28^ YAP has also been shown to play dynamic roles in compensatory cardiac hypertrophy in pressure overload condition. Genetic inhibition of fibroblast YAP alleviates cardiac dysfunction and fibrosis resulting from myocardial infarction,^29^ indicating that targeting YAP in fibroblasts holds promise as a therapeutic approach to prevent fibrotic remodeling and HF. TEAD1 deletion in CMs leads to mitochondrial dysfunction^13^ and lethal dilated cardiomyopathy.^11,12^ So far, nearly all cardiac studies involving TEAD1 have focused on its role in CMs. Vivek et al. has demonstrated TEAD1 might play a crucial role in transcriptional regulation during the process of cardiac fibroblasts (CFs) changing into induced cardiomyocytes (CMs), which is essential for their generation,^30^ however, a more complete understanding of the mechanisms underlying CFs differentiation remains elusive.

Wnt family proteins activate the Wnt signalling pathway to regulate embryonic development and the cell cycle.^31^ Our findings revealed the Wnt signalling pathway as the most significant category. RNA-seq and ChIP-seq revealed Wnt4 as a downstream target of TEAD1. Wnt4 plays a critical role in skin, renal, and cardiac development.^32,33^ Furthermore, its activation occurs in instances of fibrotic injury, including those associated with cardiovascular disease, chronic kidney fibrosis, and skin wounds.^34–36^ Wnt4 facilitates the differentiation of myofibroblasts in interstitial pericytes and fibroblasts through the canonical Wnt/β-catenin signalling pathway during renal fibrosis.^37^ Our research offers fresh perspectives on unraveling the mechanism underlying the role played by Wnt4 in TEAD1-regulated CFs differentiation.

The TEAD1 motif stands out prominently among sequences captured by cardiac transcription factors p300, GATA4, and MEF2A40.^38^ This is not surprising given that eukaryotic transcription factors typically bind to distant enhancers and, through intricate multi-protein complexes, form loops to reach the transcription start site (TSS) to modulate gene transcription.^39^ BRD4 is the most studied member of the bromodomain and extra-terminal domain (BET) family and has been established as a key cardiac transcription cofactor that plays a crucial role in cardiac remodeling and HF pathogenesis.^40–43^ McKinsey and colleagues have demonstrated that the association of BRD4 with specific gene enhancers in fibroblasts can be modified by TGF-β receptor signaling and provided explicit evidence to support a critical role of TEAD1 in CFs differentiation.^44^ A bromodomains (BDs) -dependent interaction between BRD4 and p65/RelA-K310ac have been implicated in innate immunity and similar interactions with hematopoietic TFs are essential for acute myeloid leukaemia pathogenesis.^45,46^ Therefore, the downregulation of Wnt4 expression after JQ1 administration may indicate that these two BDs (BD1 and BD2) are required for the formation of the TEAD1/BRD4 complex, suggesting a mechanism by which a widely distributed chromatin coactivator (BRD4) can be selectively directed to particular genomic sites through its interaction with a DNA-binding transcription factor (TEAD1).

TEAD1 is the major mediator of YAP in the Hippo pathway and the dysregulated YAP-TEAD1 activity is associated with various diseases, especially in cardiac remodeling.^28,29,47^ Recently, the development of drugs targeting Hippo pathway molecules, especially the YAP1/TAZ-TEAD transcription complex, has been vigorously pursued.^18,48,49^ Developing a therapeutic strategy that selectively disrupts the interaction between YAP and TEAD1 holds promise but presents a challenging endeavor. In 2021, Tang et al. reported that VT103 appears to block recombinant TEAD1 auto-palmitoylation and reduces YAP interaction with TEAD1 but not TEAD4.^19^ Hence, a selective TEAD1 inhibitor VT103 was used to evaluate the protective effect in vitro and in vivo. We showed that TEAD1 inhibitor VT103 administration showed similar beneficial effects as TEAD1 deficiency in CFs after pressure overload.

There are several shortcomings in our study. First, we failed to provide evidence that TEAD1/BRD4 signaling is responsible for the beneficial effect of knocking out TEAD1 in CFs in vivo. We believe that constructing fibroblast-overexpressing TEAD1 mice and then using pharmacologic inhibitor of BRD4 (JQ1) in the future can provide a stronger evidence for the above question.

Our study identified fibroblast-derived TEAD1 as an essential regulator of pathological cardiac remodeling in mice. Moreover, our findings strongly suggested the TEAD1/BRD4-Wnt4 signaling pathway as a promising therapeutic strategy for the treatment of pathological cardiac remodeling and HF.

## Materials and Methods

### Human samples

The experiments conformed to the principles set out in the WMA Declaration of Helsinki and the ethical review committee of Zhongshan Hospital, Fudan University (B2022-267R). All participants provided written informed consent at the time of enrolment. Human myocardial specimens were collected from the left ventricle-free wall of explanted hearts of non-ischemic hypertrophic cardiomyopathy (HCM) hearts (*n* = 10) and the healthy heart samples (*n* = 8) were obtained from brain-dead donors with normal circulatory supply following the guideline of China Transplant Services. Detailed clinical characteristics of patients is provided in supplementary table 2.

### Animals

Male C57BL/6 J mice aged 8 weeks (weight 20–23 g) were purchased from Cyagen Biosciences Inc (Guangzhou, Guangdong, China). All experiments were conducted randomly, and investigators were blinded through numerical coding of mice and samples. All animal experiments were performed in accordance with the standards of the ARRIVE guidelines and were reviewed and approved by the Animal Care and Use Committee at Zhongshan Hospital, Fudan University, China.

### AAV9 production and injection

3XFlag-tagged Wnt4 and ZsGreen were subcloned into ITR-containing AAV9 (AAV9-Tcf21) vector that harbors CFs-specific transcription factor 21 promoter (Tcf21) to generate AAV9-Tcf21-Wnt4-3xFlag-ZsGreen and AAV9-Tcf21-ZsGreen. To produce the virus particles, they were transfected to HEK293T cells together with pAAV-RC (encoding Rep/Cap) and pHelper plasmids using polyethylenimine. Cells were collected and resuspended in lysis buffer (20 mM Tris pH 8.0, 150 mM NaCl, 1 mM MgCl2) with 250 U/ml Benzonase (Sigma E8263) 72 h post transfection. AAV9 viruses were purified using AAV Purification Maxiprep Kit (Biomiga, V1469-01) according to the manufacturer’s instructions. qRT-PCR was used to determine the viral titer by measuring the copies of viral genome. The concentrations of AAV9-Tcf21-Wnt4-3xFlag-ZsGreen and AAV9-Tcf21-ZsGreen were 1.7×10^12^vg/mL and 1.4×10^12^vg/mL, respectively. The concentrated AAV9 viruses were suspended in PBS, aliquoted and stored at -80 °C until use. Each mouse (20–24 g) was injected with 1×10^11^ vg through the route of tail vein.

### RNA-sequencing and data analysis

Total RNA was extracted using the TRIzol reagent according to the manufacturer’s protocol. RNA purity and quantification were evaluated using the NanoDrop 2000 spectrophotometer (Thermo Scientific, USA). RNA integrity was assessed using the Agilent 2100 Bioanalyzer (Agilent Technologies, Santa Clara, CA, USA). Then the libraries were constructed using TruSeq Stranded mRNA LT Sample Prep Kit (Illumina, San Diego, CA, USA) according to the manufacturer’s instructions. The transcriptome sequencing and analysis were conducted by OE Biotech Co., Ltd. (Shanghai, China).

### Statistical Analysis

For statistical analysis, data are presented as mean ± standard deviation (SD). Normality of the distribution of data was assessed using the Shapiro-Wilk test. Before *t*-test and ANOVA, we firstly test the homogeneity among variances. If it shows the variances are unequal, we then use Welch’s *t*-test and Welch’s ANOVA followed by Dunnett’s T3 post hoc test for analysis. Differences of two groups were evaluated using unpaired two-tailed Student’s *t*-test when normal distribution was satisfied. Statistical differences among>2 groups were analyzed by one-way analysis of variance (ANOVA) or two-way ANOVA (if there were 2 factors) followed by Bonferroni post hoc analysis for data with normal distribution. All statistical analyses were performed with Prism software (GraphPad prism for windows, version 9.0, Nashville, USA). *P* values less than 0.05 was considered statistically significant.

### Supplementary information

supplementary material

## Data Availability

All the datasets presented in the paper are available from the corresponding author upon reasonable request.
