# Supplementary material for: TEA domain transcription factor 1 (TEAD1) induces cardiac fibroblasts cells remodeling through BRD4/Wnt4 pathway
Source: Signal Transduct Target Ther. 2024 Feb 19;9:45. doi: 10.1038/s41392-023-01732-w (PMC10876703; doi:10.1038/s41392-023-01732-w)
Supplement: Supplementary file 1 — supplementary material [file 41392_2023_1732_MOESM1_ESM.docx]

Supplementary Materials for

TEA domain transcription factor 1(TEAD1) induces Cardiac Fibroblasts cells Remodeling through BRD4/Wnt4 Pathway

Shuai Song†, Xiaokai Zhang†, Zihang Huang†, Yongchao Zhao†, Shuyang Lu, Linqi Zeng, Fengze Cai, Tongyao Wang, Zhiqiang Pei, Xinyu Weng, Wei Luo, Hao Lu, Zilun Wei, Jian Wu, Peng Yu, Li Shen, Xiaochun Zhang, Aijun Sun*, Junbo Ge*

† These authors contributed equally to this work.

Correspondence to: Aijun Sun, email: [sun.aijun@zs-hospital.sh.cn](mailto:sun.aijun@zs-hospital.sh.cn) or Junbo Ge, email: [jbge@zs-hospital.sh.cn](mailto:jbge@zs-hospital.sh.cn)

**This PDF file includes:**

Materials and Methods

Figures. S1 to S15

Tables S1 to S8

**Materials and Methods**

**Generation of knockout of TEAD1 in cardiac fibroblasts and myofibroblast mice**

Conditional transgenic mice targeting TEAD1 gene (TEAD1^fl/fl^) were generated by Cyagen Biosciences Inc (Guangzhou, Guangdong, China) in the C57BL/6J background. Briefly, 14 exons were identified and exon 3 was selected as conditional knockout region (CKO region). The knockout of Exon 3 resulted in frameshift and loss of function of the mouse TEAD1 gene. The col1a2-Cre/ERT mice, containing a Cre-ERT recombinase gene were purchased from JAX Lab (#029567)^1^. The postn-MerCreMer mice, containing tamoxifen-inducible cre recombinase from the mouse periostin gene were purchased from JAX Lab (#029645)^2^. TEAD1^fl/fl^ mice were bred with col1a2-Cre/ERT mice and postn-MerCreMer mice to achieve fibroblast-specific and myofibroblast-specific TEAD1 knockout in the adult. Mice were intraperitoneally treated with 20mg/kg of tamoxifen dissolved in corn oil for 5 days at 8-10 weeks of age. Mice of different lines (TEAD1^fl/fl^, Col1a2-Cre, and Postn-Cre) were genotyped with polymerase chain reactions (PCR). PCR for genotyping was performed with primers: 5’- ACAAATCAAGGTCTCCTCTTGAGC-3’(sense) and 5’-AAAGGCAGACTCCTTCATTGGAA-3’(antisense), which yielded 217 bp products for TEAD1^fl/fl^; 5’- TCCAATTTACTGACCGTACACCAA-3’(sense) and 5’- CCTGATCCTGGCAATTTCGGCTA -3’(antisense), which yielded 500 bp products for col1a2. 5’- GGTGCTTCTGTAAGGCCATC-3’(sense) and 5’- GGTGGGACATTTGAGTTGCT -3’(antisense), which yielded 270 bp products for postn.

**Transverse Aortic Constriction**

A mouse model for cardiac pressure overload was established through transverse aortic constriction (TAC) surgery performed on 8-week-old WT mice or TEAD1 cKO mice, following the previous described procedure.3. Briefly, mice were anesthetized using intraperitoneal injection of pentobarbital sodium at a dosage of 50 mg/kg.. The trachea of mouse was exposed through midline cervical incision, and the thymus was pushed aside.Following anesthesia, a trans-sternal thoracotomy was conducted, extending from the suprasternal notch to the second rib, exposing the aortic arch. Using a 7-0 silk thread ligature, the narrowing of the aortic arch was achieved by tying it against a 27-gauge de-sharpened needle positioned between the innominate and the left common carotid artery. The needle was then removed with a stenosis left in the aortic lumen. Sham-operated mice underwent an identical procedure, excluding the aortic constriction step.. The mice were continuously monitored until recovery after surgery. Each investigator was blind to the allocation of treatment and genotype.

**Ang-II infusion**

Mouse model of neurohumoral stimulation was generated by implantation of osmotic mini-pumps on 8-week-old WT mice or TEAD1 cKO mice as described previously^4^. Concisely, mice were anesthetized via intraperitoneal injection of pentobarbital sodium (50 mg/kg), followed by the subcutaneous implantation of osmotic mini-pumps (Alzet, 2004, USA) to infuse Ang-II (Sigma, A9525, 750 ng/kg/min). These pumps were placed in the back region and remained implanted for 28 days. Control animals received an infusion of saline. Systolic blood pressure (SBP) was measured at second day following implantation via tail-cuff method using the BP-2010A. Echocardiography was performed at 28 days before killing mice. An investigator was blind to the allocation of treatment and genotype.

**Echocardiography Analysis**

Cardiac structure and function were assessed by transthoracic echocardiography (VisualSonics VeVo 2100 Imaging System, Toronto, Canada) equipped with a 30-MHz linear transducer. Mice were anesthetized using isoflurane (1%-2%) and subsequently placed on a heated pad to sustain body temperatures within the range of 36.9℃ to 37.3℃. The heart rates were maintained at around 500 bpm. Standard long-axis M-mode measurements were recorded. Systolic and diastolic LV posterior wall (LVPW) thicknesses, interventricular septum (IVS) thicknesses, ejection fraction (EF), fractional shortening (FS), left ventricular end-diastolic dimension (LVEDD), left ventricular end-systolic volume (LVESV) were measured for each mouse at the target heart rate. The subsequent analysis was conducted by an investigator who was blind to the allocation of treatment and genotype.

**Neonatal Mice Cardiac Fibroblasts Culture**

Neonatal mice cardiac fibroblasts (NMCFs) were isolated as previously described with some modifications^5^. Briefly, 1-3-day-old neonatal C57BL/6J mice were executed and their hearts were harvested immediately. Then these hearts were washed with cold Hank's balanced salt solution (HBSS) to remove blood cells. Ventricles were minced into pieces and transferred to trypsin (0.01 mg/ml, Gibco, 15050065) in HBSS at 4°C overnight. The next day, predigested hearts fragments were treated with 1500 Units/mL collagenase type II (Worthington, LS004176) for another 17 minutes at 37°C under constant shaking. Subsequently, the digested cell suspension was gently triturated by pipetting and then passed through a 70 μm cell strainer to eliminate tissue debris. Finally, all the digested cells were centrifuged, resuspended in DMEM with high glucose (Gibco, 11965092) with 10% fetal bovine serum (FBS, Biological Industries), and then seeded into 10cm plates. After incubation at 37 ℃ with 5% CO_2_ for 1.5h, the supernatant was deprived and the cells adherent to the bottom of plates were CFs.

**Adult mouse cardiomyocytes, endothelial cells and fibroblasts isolation**

Adult cardiomyocytes (CMs), endothelial cells (ECs) and fibroblasts were isolated from the ventricles of wild-type male mice as previously described with some modifications^6^. Briefly, mice were anesthetized, and the heart was exposed. Then 7ml EDTA buffer (130 mM NaCl, 5 mM KCl, 0.5 mM Na_2_HPO_4_, 10 mM HEPES, 10 mM Taurine, 10 mM D-glucose, 10 mmol/l BDM, 5mM EDTA, pH 7.8) was injected steadily into the right ventricle after cutting the descending aorta. The heart was then excised and sequentially perfused with 10ml EDTA buffer, 3ml perfusion buffer (130 mM NaCl, 5 mM KCl, 0.5 mM Na_2_HPO_4_, 10 mM HEPES, 10 mM Taurine, 10 mM D-glucose, 10 mmol/l BDM, 1mM MgCl_2_, pH 7.8) and 30ml pre-warmed collagenase buffer (perfusion buffer with 0.5 mg/ml collagenase II, 0.5 mg/ml collagenase IV and 0.05 mg/ml protease XIV). All the buffer was filtered through a 0.22-μm filter before used. Cardiac ventricles were further dissociated into pieces in collagenase buffer. Once digestion was halted using 5 mL of stop buffer (comprising perfusion buffer with 5% FBS), the resulting cell suspension was filtered through a 100-μm filter into a 50 mL tube, allowing the formation of a pellet through gravity settling. The pellet with CMs underwent 3 steps of calcium reintroduction and were plated on laminin coated culture dishes. The remaining cellular components was collected and then filtrated through 70 μm strainers to obtain non-cardiomyocyte fraction. The supernatant was incubated with CD45 MicroBeads (Cat No. 130-052-301, Miltenyi Biotec) (1×10⁷ total cells per 10 μL MicroBeads) for 15 minutes at 4 °C to deplete the CD45^+^ cells. The CD45^-^ fraction was then incubated with CD31 MicroBeads (Cat No. 130-097-418, Miltenyi Biotec) (1×10⁷ total cells per 10 μL MicroBeads) for 15 minutes at 4°C and purified through the LD Column (Cat No. 130-042-901, Miltenyi Biotec) according to the manufacturer’s instructions. The unlabeled cells that pass through the column was CFs (CD45^-^/CD31^-^), and the magnetically labeled cells was ECs (CD45^-^/CD31^+^).

**siRNA transfection**

TEAD1 or Wnt4 specific siRNA and negative control siRNA sequences were designed and synthesized by RiboBio Co., Ltd. Three target sequences of TEAD1 siRNA used in mice CFs were as follows: TEAD1 siRNA (1) GCGGACTTAAACTGCAATA; TEAD1 siRNA (2) TTGGGAAACAAGTAGTAGA; TEAD1 siRNA (3) CAAGCTCAAACACCTACCA. Target sequence of Wnt4 siRNA used in mice CFs was as follows: GCAGGTGCAGATGTGCAAA. Target sequence of YAP siRNA used in mice CFs was as follows: CGAGATGAGAGCACAGACA. siRNA transfection was performed as the instruction of Lipofectamine® RNAiMAX Reagent (Invitrogen, 13778075). At the culmination of a 48-hour period following siRNA transfection, cells were gathered and subjected to analysis via qRT-PCR and western blotting.

**Adenovirus infection**

For recombinant adenovirus construction, the TEAD1 or BRD4 cDNA, alongside the green fluorescence protein gene (Ad-GFP; control) cloned by PCR was inserted into pDC315-EGFP vector (purchased from Hanbio Co. Ltd, Shanghai, China) respectively, under the control of the mouse cytomegalovirus (CMV) promoter. To generate the recombinant adenoviruses, the pDC315-X and pBHGlox E1,3Cre were co-transfected into HEK293T cells using Lipofectamine^TM^ 3000 Reagent (Invitrogen, L3000001). Ad-TEAD1 or Ad-BRD4 and Ad-GFP were propagated in HEK293T cells. The propagated recombinant adenoviruses within the HEK293T cells underwent purification, and the virus titer was quantified using plaque assays. The stock solutions of Ad-TEAD1, Ad-BRD4 and Ad-GFP were 1.24×10^10^, 1.37×10^10^ and 1.53×10^10^ plaque formation unit (PFU)/ml, respectively. CFs were infected with indicated adenovirus particles at MOI of 50.

**Transwell migration assay**

The migration assay was conducted utilizing a 24-well microchemotaxis chamber (Corning, 8.0μm, 3422), following established protocols as described previously^4^. In brief, different reagents (si-NC, si-TEAD1, Ang-II, Ang-II+si-TEAD1, OE-NC and OE-TEAD1) in DMEM supplemented with 0.5% BSA were placed in the lower wells. CFs were subjected to digestion using 0.25% trypsin, followed by two washes with PBS. Subsequently, these cells were resuspended in the same medium at a concentration of 1×10^5 cells/ml. The prepared cells were then introduced into the upper wells, which were partitioned from the lower wells by 8 μm-pore polyvinylpyrrolidone-free polycarbonate filter membranes. After incubation at 37 °C and 5% CO_2_ for 48 h, the cells that migrated to the outer side were washed using PBS, followed by fixation using 4% formalin for 15 minutes. Subsequently, they were stained with 0.25% crystal violet for 15 minutes, rinsed with sterile water, air-dried, and finally mounted. The migrated cells were photographed and collected to count the number of migrating cells in each field.

**Collagen lattice contraction assays**

Collagen lattice contraction assays were carried out using CFs. Collagen lattices were created by blending cells with a neutralized solution of rat tail collagen type I (Corning, 354236, 3.34 mg/ml). A 0.2% acetic acid solution is prepared using 100% glacial acetic acid, subsequently sterilized by filtering it through a 0.2-μm filter, and cooled to 4℃. Following sterile conditions, type 1 collagen is combined with the 0.2% acetic acid solution to create a 3 mg/mL collagen solution. To identify NaOH titration of Collagen, the optimal amount of NaOH (8ul) to add to the collagen/media mixture, use the same quantity for all subsequent gels. The contraction assays utilized a final collagen concentration of 3.0 mg/ml and a cell concentration of 1×10^5^ cells/ml within the matrix. Each experiment was conducted at least thrice independently, and for every set of conditions, the experiment was repeated three times.

**Histopathology analysis**

The hearts of the mice were perfused with cold PBS and excised. Then the hearts were fixed in 4% paraformaldehyde for 48h and embedded in paraffin. For hematoxylin and eosin (H&E) staining, the hearts were sectioned longitudinally, and the staining was performed according to the manufacturer's protocols of the H&E buffer (Servicebio, G1005). Picrosirius red (PSR) staining was used to assess collagen deposition. Heart sections were transversely obtained at 6 μm intervals and the slides were incubated with Sirius Red solution (Solarbio, G1018) at room temperature for 1h. The fibrotic area in each field was quantified using NIH ImageJ software. For quantification, total fibrosis was averaged from randomized 3 fields per section and 3 heart sections per mouse. Each group contains 4-10 mice. To detect the parameters of hypertrophic growth, the slides were incubated with FITC-conjugated wheat germ agglutinin (WGA, W11261, Invitrogen) for 15min at room temperature. 4,6-Diamidino-2-phenylindole (DAPI) was used for the nuclear staining. The cardiomyocyte cross-sectional areas were photographed using Zeiss fluorescence microscope with captured images. For immunofluorescence, frozen heart sections or CFs were were fixed in 4% paraformaldehyde, being permeabilized and blocked with 0.3% Triton-X100 with 3% BSA in PBS for 1 h. After that, the heart sections and cells were incubated with primary antibody against α-SMA (abcam, ab124964), Col1a1 (abcam, ab260043) and Col3a1 (Santa Cruz, sc-271249) and then incubated with Alexa Fluor® 647 or 488 conjugated secondary antibody. After rinsing with PBS, 4,6-Diamidino-2-phenylindole (DAPI) was used for the nuclear staining. Finally, a laser scanning confocal microscope (Lecia, Wetzlar, Germany) was used to acquire immunofluorescence staining images and the fluorescence intensity was quantified by Image-Pro Plus software (version 6.0) and was analyzed by a person blinded to treatment.

**Western Blotting**

Proteins were extracted from tissues or cultured cells as described previously. The equal amounts of protein extracts were separated on 10-15% SDS-polyacrylamide gels by electrophoresis and then transferred to poly vinylidene fluoride (PVDF) membranes (Merck, #ISEQ00010). The membranes underwent blocking with 5% non-fat milk in Tris Buffered Saline with Tween 20 (TBST) for 1 hour at room temperature. Subsequently, they were individually incubated with primary antibodies overnight at 4℃. Following washes with TBST, the membranes were exposed to horseradish peroxidase (HRP)-conjugated anti-rabbit or anti-mouse secondary antibodies for 1 hour at room temperature. The specific bands were visualized and detected using Electrochemiluminescence (ECL) substrate (Thermo, #32132), and images were obtained by ChemiDoc™ Imaging System (Bio-Rad, CA, US). The density of the protein blots was quantified by NIH ImageJ software after normalization to β-actin. Primary antibodies used were listed in Supplementary table 8.

**Quantitative Real-Time PCR**

Total RNA from tissues or cultured cells was extracted using RNAiso Plus (Takara, 9109) according to the manufacturer’s instructions. The concentration and purity of RNA were assessed using a Nanodrop spectrophotometer. Subsequently, 1000 ng of total RNA, possessing an A260/A280 ratio within the range of 1.8-2.0, was subjected to reverse transcription using the PrimeScript RT Master Mix (Takara, #RR036A) to generate complementary DNA (cDNA). Then quantitative real-time polymerase chain reaction (qRT-PCR) was performed using SYBR Green dye (TaKaRa, #RR820A) on a CFX96 real-time PCR System (Bio-Rad Laboratories, Inc., CA, USA). A total of 10 μL reaction system was used, including DNA template 1 μL, SYBR Green 5 μL, forward primers 0.5 μL, reverse primers 0.5 μL and ddH_2_O 3 μL. The specific primers used in this study were listed in supplementary table 1. The PCR program was set as follows: 30 s at 95°C, 5 s at 95°C, and 30 s at 60°C for 35 cycles. Relative quantitation of gene expression was normalized to β-actin by using the standard 2^−ΔΔCT^ method.

**Coimmunoprecipitation**

For separation of cytosolic and nuclear fractions, cells or tissues were washed with cold PBS once and scraped with lysis buffer A (10 mM HEPES, 0.05% NP-40, 10 mM KCl, 0.5 mmol/L DTT, 1.5 mM MgCl_2_ and cocktail 100×). The generated homogenate was oscillated and centrifuged for 13,000 rpm, 1 min at 4°C. The supernatant was collected as the cytosolic fraction. After that, the pellet was further lysed with lysis buffer B (20 mM HEPES, 25% Glycerol, 0.42 M NaCl, 0.2 mM EDTA, 0.5 mmol/L DTT, and cocktail 100×) and oscillated every 5 min for 30 min on the ice. Then the homogenate was centrifuged for 13,000 rpm for 10 min at 4°C and the supernatant was reserved as the nuclear fraction. Nuclear pellet was lysed in IP buffer (20mM Tris pH 7.5, 150mM NaCl, 1% Triton-X 100, cocktail 100×) with incubation of protein G Dynabeads (Invitrogen, 10004d) for precleaning for 1 h on a revolver at 4 °C. Then the pre-cleared lysates were incubated with 5 μg TEAD1 (Abcam, ab133533), BRD4 (Abcam, ab128874) and YAP (CST, 14074) antibody on a rotator at 4 °C overnight. The next day, the protein G Dynabeads were added to the lysates and incubated for 3 h at 4 °C. After three times washes in IP buffer, beads were resuspended in 40 μL of 2× loading buffer and boiled at 95 °C for 10 mins to separate the protein and beads. Following the procedures, the samples were analysed using immunoblotting techniques.

**Luciferase assays**

For the construction of Wnt4 promoter luciferase reporter assay, a fragment containing the mouse Wnt4 promoter region (1000bp relative to the transcription start site of the Wnt4 gene) was amplified by PCR. The fragment was synthesized and inserted between the KpnI and XhoI loci of pGL3-Basic luciferase reporter plasmid. For determination of the effect of TEAD1 knockdown or overexpression and BRD4 inhibition with JQ1 or overexpression on Wnt4 promoter, cardiac fibroblasts (CFs) were transfected with si-TEAD1 or TEAD1^OE^ and JQ1 or BRD4^OE^. CFs were transfected with 0.5μg of the Wnt4 promoter luciferase reporter plasmid and 0.1 μg of the Renilla luciferase plasmids with Lipofectamine™ 3000 Transfection Reagent (Invitrogen, L3000001). 24h after transfection, cells were harvested and the luciferase activity was detected with a dual luciferase reporter assay system (Promega, USA). Renilla luciferase activity was normalized based on firefly luciferase activity.

**Chromatin Immunoprecipitation (ChIP) Assays and sequencing**

ChIP-qPCR was performed as described previously with minor modifications^7^. Briefly, chromatin in CFs was harvested and cross-linked with 1% formaldehyde for 10 min at room temperature and stopped by freshly prepared 0.125mol/L Glycine. Cross-linked chromatin complexes were subsequently lysed using Nuclei Isolation Buffer (20 mM HEPES pH 7.5, 10 mM KCl, 1 mM EDTA, 0.2% NP40, 10% Glycerol, 1× Protease Inhibitor Cocktail (PIC; Roche)) and then sonicated in Sonication Buffer (20 mM Tris Cl pH 8.0, 2 mM EDTA, 150 mM NaCl, 1% NP40, 0.1% SDS, 1× PIC) using a Qsonica Q125 (80% amplitude, pulse for 20s on and 10s off for a total sonication “on” time of 15 mins of elapsed time; Qsonica, Q125, America). After sonication, immunoprecipitation was performed with TEAD1 (Abcam, ab133533) and BRD4 (Abcam, ab128874) or IgG (CST, catalog no. 2729, 1:200) antibody at 4 °C overnight. Bead-bound DNA was washed with three times in RIPA Buffer (1% TritonX-100, 1% NP40, 50 mM HEPES pH 8.0, 1 mM EDTA, 0.7% sodium deoxycholate, 1× PIC), de-crosslinked in a 65 °C water bath overnight. The eluted DNA was incubated with RNase A (Invitrogen, EN0531) and proteinase K (Beyotime, ST535) to remove RNA and protein. Following the washing of the immunoprecipitated complex, DNA extraction and purification were carried out using the QIAquick PCR Purification kit from QIAGEN (28104).. The following primers were used for ChIP-qPCR of targeting genes:

Mice Wnt4, forward: 5'- CGCTTAAACCTAAGAGTGGCC-3'

Mice Wnt4, reverse: 5'- TGACCACATCTCTTTGGCCC-3';

Mice GAPDH-TSS, forward: 5'- CCACTAGGCGCTCACTGTTC-3'

Mice GAPDH-TSS, reverse: 5'- CGCGAACTCACCCGTTGAC-3';

Mice BLANK, forward: 5'- TGCCCAGCCTCAGTTTCTTA-3'

Mice BLANK, reverse: 5'- GCAACCAAACCATGAGCTGA-3';

For ChIP-seq, extracted DNA was ligated to specific adaptors followed by deep sequencing in the Illumina Novaseq 6000 using 150bp paired-end. The initial processing of raw data in fastq format involved the utilization of in-house perl scripts. This step aimed to generate clean data (clean reads) by eliminating reads containing adapters, reads with ploy-N sequences, and low-quality reads from the raw data. Simultaneously, calculations were performed to determine Q20, Q30, and GC content data. Subsequent analyses were conducted solely on the clean data possessing high quality. The adaptor sequences were removed before read mapping. The clean reads were then aligned to reference genome sequences using the bwa program. The bam file generated by the unique mapped reads as an input file, using macs2 software for callpeak with cutoff qvalue < 0.05. The Motif analysis was conducted using HOMER's findMotifsGenome.pl tool. The input comprised the peak file and the genome fasta file. The tool extracted DNA sequences based on the peak file, subsequently comparing these sequences with the Motif database to acquire the specific motifs. Peaks were annotated by using homer's annotatePeaks.pl. Count the results of the annotations and plot the distribution results using R. Gene ontology (GO) analysis was performed to facilitate elucidating the biological implications of unique genes in the significant or representative profiles of the gene in the experiment. Pathway analysis was used to find out the significant pathway of the genes according to KEGG database. Differential accessible peak was analyzed using DESeq2. Differentially accessible region was determined if the absolute value of the log2 fold change was 1 at a pvalue < 0.05. We thank Jiayin Biotechnology Ltd. (Shanghai, China) for the assistance with ChIP-seq assay and data analysis.

**Coimmunoprecipitation Mass spectrometry analysis**

Mass spectrometry analysis was performed as described previously^8^. The protein sample was denatured by 2% SDS buffer containing 50mM DTT for 20min RT and then boiled at 100 °C for 5min. The protein sample was alkylated for 1 hour at room temperature in the dark by addition of a final concentration of 200mM iodoacetamide (IAA). The eluted peptides were lyophilized using a SpeedVac (ThermoSavant) and resuspended in 1% formic acid 5% acetonitrile. The peptides were re-dissolved in solvent A (A: 0.1% formic acid in water) and analyzed by Orbitrap Exploris 480 with a FAIMS coupled to an EASY-nanoLC 1200 system (Thermo Fisher Scientific, MA, USA). Raw Data of DIA were processed and analyzed by Spectronaut 14 (Biognosys AG, Switzerland).

**Figure. S1. TEAD1 expression is increased in human and mouse remodeling hearts.**

**a.** Quantitative real time polymerase chain reaction (qRT-PCR) analyses of TEAD1 mRNA levels in heart samples from non-HCM and HCM patients (n=8 for non-HCM and n=10 for HCM group). **b.** Western blot and quantification of the protein levels of TEAD1 in isolated CMs after 4 weeks of TAC surgery (n=4 per group). **c.** Representative immunofluorescence images of TEAD1 and α-SMA staining in the hearts from WT mice after sham or TAC surgery (n=4 per group; scale bar=20 μm). For all statistical plots, the data are presented as mean ± SD. ns. indicates no significance between the 2 indicated groups. a and c by Welch's t-test. B by two-tailed unpaired Student’s t-test. HCM, hypertrophic cardiomyopathy.

**Figure. S2. Generation of TEAD1 deficiency in CFs in mice.**

**a**. Schematic diagram for generation of cardiac fibroblasts (CFs) specific TEAD1 deficiency mouse line. TEAD1 conditional knockout by crossing TEAD1^flox/flox^ mice with CFs specific col1a2-CreERT line. **b**. PCR analysis of DNA isolated from tails in WT, TEAD1^flox/+^, TEAD1^flox/flox^ and col1a2^+^ mice. The PCR primers used for genotype identification of TEAD1^flox/flox^ and col1a2-CreERT transgenic mice. **c, d**. TEAD1^fl/fl^col1a2^+^ mice exhibit significant reduction of TEAD1 expression in cardiac heart samples by qRT-PCR (**c**) and western blot (**d**) following tamoxifen compared with TEAD1^fl/fl^ controls (n=4 per group). **e, f**. TEAD1^fl/fl^col1a2^+^ mice exhibit significant reduction of TEAD1 expression in CFs and no significant change in ECs and CMs by qRT-PCR (**e**) and by western blot (**f**) following tamoxifen compared with TEAD1^fl/fl^ controls (n=4 per group). For all statistical plots, the data are presented as mean ± SD. ns. indicates no significance between the 2 indicated groups. c, d, ECs and CMs in e and f by two-tailed unpaired Student’s t-test; CFs in e and f by Welch's t-test. m, marker; CKO, conditional knockout, CMs, cardiomyocytes; CFs, cardiac fibroblasts; ECs, endothelial cells.

**Figure. S3. Deficiency of TEAD1 in CFs attenuates Ang-II-induced cardiac remodeling.**

**a**. Schematic for echocardiography and sample collection from 4 groups: TEAD1^fl/fl^ and TEAD1^fl/fl^col1a2^+^ were infused with saline or Ang-II (750ng/kg/day) for 4 weeks (n=4-10 per group). **b**. left ventricular EF assessed by echocardiography of TEAD1^fl/fl^ and TEAD1^fl/fl^col1a2^+^ mice 4 weeks after saline or Ang-II infusion (n=4-10 per group). **c-d**. Heart sections were stained with picrosirius red (**c**) or WGA (**d**) from TEAD1^fl/fl^ and TEAD1^fl/fl^col1a2^+^ mice infused with saline or Ang-II (n=4-10 per group; for WGA staining, scale bar=20 μm; for picrosirius red staining, scale bar=50 μm). **e**. Western blot and quantification of α‑SMA and Galectin-3 protein levels in the heart homogenates extracted from TEAD1^fl/fl^ and TEAD1^fl/fl^col1a2^+^ mice infused with saline or Ang-II (n=4-10 per group). **f**. qRT-PCR analyses of the mRNA levels of ANP, BNP and β-MHC in heart samples from TEAD1^fl/fl^ and TEAD1^fl/fl^col1a2^+^ mice infused with saline or Ang-II (n=4-10 per group). For all statistical plots, the data are presented as mean ± SD. ns. indicates no significance between the 2 indicated groups. ­b-e by two-way ANOVA with Bonferroni multiple comparison test. f by two-way ANOVA with Dunnett's T3 post hoc analysis.

**Figure. S4. Generation of myofibroblast-specific TEAD1 deficiency mice.**

**a**. Schematic diagram for generation of myofibroblast specific TEAD1 deficiency mouse line. TEAD1 conditional knockout by crossing TEAD1^flox/flox^ mice with myofibroblast specific postn-MerCreMer line. **b**. The PCR primers used for genotype identification of TEAD1^flox/flox^ and postn-MerCreMer transgenic mice. **c, d**. TEAD1^fl/fl^postn^+^ mice exhibit significant reduction of TEAD1 expression in cardiac heart samples at 13^th^ day after TAC by qRT-PCR (**c**) and western blot (**d**) following tamoxifen compared with TEAD1^fl/fl^ controls (n=4 per group). **e, f**. TEAD1^fl/fl^postn^+^ mice exhibit significant reduction of TEAD1 expression in myofibroblasts (MFs) and no significant change in ECs and CMs at 13^th^ day after TAC by qRT-PCR (**e**) and western blot (**f**) following tamoxifen compared with TEAD1^fl/fl^ controls (n=4 per group). **g, h**. TEAD1^fl/fl^postn^+^ mice exhibit significant reduction of TEAD1 expression in cardiac heart samples at 13^th^ day after Ang-II infusion by qRT-PCR (**g**) and western blot (**h**) following tamoxifen compared with TEAD1^fl/fl^ controls (n=4 per group). **i, j**. TEAD1^fl/fl^postn^+^ mice exhibit significant reduction of TEAD1 expression in MFs and no significant change in ECs and CMs at 13^th^ day after Ang-II infusion by qRT-PCR (**i**) and western blot (**j**) following tamoxifen compared with TEAD1^fl/fl^ controls (n=4 per group). For all statistical plots, the data are presented as mean ± SD. ns. indicates no significance between the 2 indicated groups. c, d, g, h, ECs and CMs in e, f, i and j by two-tailed unpaired Student’s t-test; MFs in e, f, i and j by Welch's t-test. m, marker; CKO, conditional knockout, CMs, cardiomyocytes; MFs, myofibroblasts; ECs, endothelial cells.

**Figure. S5. Myofibroblast-specific deficiency of TEAD1 attenuates Ang-II-induced cardiac remodeling.**

**a**. Schematic for echocardiography and sample collection from 4 groups: TEAD1^fl/fl^ and TEAD1^fl/fl^postn^+^ were infused with saline or Ang-II (750 ng/kg/day) for 4 weeks (n=4-10 per group). **b**. left ventricular EF assessed by echocardiography in TEAD1^fl/fl^ and TEAD1^fl/fl^postn^+^ mice after 4 weeks saline or Ang-II infusion (n=4-10 per group). **c-d**. Heart sections were stained with picrosirius red (**c**) or WGA (**d**) from TEAD1^fl/fl^ and TEAD1^fl/fl^postn^+^ mice infused with saline or Ang-II (n=4-10 per group; for WGA staining, scale bar=20 μm; for picrosirius red staining, scale bar=50 μm). **e**. Western blot and quantification of α‑SMA and Galectin3 protein levels in the heart homogenates extracted from TEAD1^fl/fl^ and TEAD1^fl/fl^postn^+^ mice infused with saline or Ang-II (n=4 per group). **f**. qRT-PCR analyses of the mRNA levels of ANP, BNP and β-MHC in heart samples from TEAD1^fl/fl^ and TEAD1^fl/fl^postn^+^ mice infused with saline or Ang-II (n=4-10 per group). For all statistical plots, the data are presented as mean ± SD. ns. indicates no significance between the 2 indicated groups. ­b-e by two-way ANOVA with Bonferroni multiple comparison test. f by two-way ANOVA with Dunnett's T3 post hoc analysis.

 **Figure. S6. The effect of VT103 on the expression of TEAD1 and YAP-TEAD interaction.**

**a.** qRT-PCR analyses of the mRNA levels of TEAD1 in CFs treated with VT103 of 0.1μM, 0.2μM, 0.5μM, 1μM, 2μM, 5μM, 10μM (n=3 per group). **b**. Western blot and quantification of TEAD1 protein levels in CFs treated with VT103 of 0.1μM, 0.2μM, 0.5μM, 1μM, 2μM, 5μM, 10μM (n=3 per group). **c**. Endogenous immunoprecipitation of TEAD1 and YAP in fibroblasts in the presence or absence of VT103 (n=3 per group). **d**. Endogenous immunoprecipitation of TEAD1 and YAP in cardiac lysates extracted from WT mice treated with VT103 (n=3 per group). For all statistical plots, the data are presented as mean ± SD. a-b by one-way ANOVA with Bonferroni multiple comparison test.

**Figure. S7. TEAD1 inhibition attenuates Ang II-stimulated cardiac fibroblast differentiation, migration, and ECM synthesis in vitro.**

1. Heatmap of differentially expressed genes in CFs stimulated with saline or Ang-II (1μM) in the presence or absence of VT103 (1μM) (n=3 per group). **b.** GO term enrichment analysis of genes downregulated in Ang-II+VT103 compared with Ang-II based on RNA-seq dataset (n=3 per group). **c.** KEGG analysis of genes downregulated in Ang-II+VT103 compared with Ang-II (n=3 per group). **d**. Representative images of immunofluorescence staining against α-SMA in CFs stimulated with saline or Ang-II in the presence or absence of VT103 (n=4 per group; scale bar=100 μm).**e**. Collagen gel contraction and gel area seeded CFs stimulated with saline or Ang-II in the presence or absence of VT103 (n=4 per group). **f**. Migration of CFs stimulated with saline or Ang-II in the presence or absence of VT103 (n=4 per group; scale bar=100 μm). **g**. Western blot and quantification of α‑SMA and Galectin-3 protein levels in CFs stimulated with saline or Ang-II in the presence or absence of VT103 (n=3 per group). For all statistical plots, the data are presented as mean ± SD. ns. indicates no significance between the 2 indicated groups. ­­­­­g by two-way ANOVA with Bonferroni multiple comparison test.­­ Ang=II, angiotensin-II; GO, Gene ontology; KEGG, Kyoto Encyclopedia of Genes and Genomes.

**­­**

**Figure. S8. The effect of TEAD1 knockdown and overexpression on the signaling pathways.**

**a**. Cartoon representation of RNA-seq strategy used on saline or Ang-II- treated CFs infected with si-TEAD1 or Ad-TEAD1. **b-c**. Heatmap of 77 genes upregulated in Ang-II-treated CFs compared with transfected with si-NC and downregulated in Ang-II+si-TEAD1 compared with Ang-II by RNA-seq analysis (**c**). Heatmap of 20 genes which were upregulated in CFs infected with adenovirus expressing TEAD1 compared with NC in the indicated 77 genes (**b**). Each column represents an individual replicate and there are 3 replicates per group. Each row represents an individual gene. **d**. GO term enrichment analysis of genes downregulated in Ang-II+si-TEAD1 compared with Ang-II based on RNA-seq dataset. **e**. KEGG analysis of genes downregulated in Ang II+si-TEAD1 compared with Ang-II si-NC based on RNA-seq dataset. **f**. Enrichment plots of GSEA comparison of Ang-II and Ang-II+si-TEAD1. **g**. Enrichment plots of GSEA comparison of OE-NC and OE-TEAD1.

**Figure. S9. Wnt4 was upregulated in human HCM samples.**

**a.** qRT-PCR analyses of TEAD1 mRNA levels in heart samples from non-HCM and HCM patients (n=8 for non-HCM and n=10 for HCM patients). **b.** Western blot of TEAD1 in heart samples from non-HCM and HCM patients (n=4 per group). For all statistical plots, the data are presented as mean ± SD. a-b by Welch's t-test.

**Figure. S10. The effect of TEAD1 overexpression on the non-canonical Wnt signal pathway.**

**a.** Western blot and quantification of TEAD1, α‑SMA, PKC and CamkII protein levels in CFs infected with adenovirus expressing TEAD1 or NC. **b.** Western blot and quantification of TEAD1, α‑SMA, p-JNK1/2 and JNK1/2 protein levels in CFs infected with adenovirus expressing TEAD1 or NC. For all statistical plots, the data are presented as mean ± SD. a and b by two-tailed unpaired Student’s t-test.

**Figure. S11. Cardiac fibroblasts- and myofibroblasts-specific deficiency of TEAD1 reduced Wnt4 expression in vivo.**

**a.** Western blot and quantification of Wnt4 protein levels in TEAD1^fl/fl^ and TEAD1^fl/fl^col1a2^+^ mice after sham or TAC surgery for 4 weeks (n=4 per group). **b.** Western blot and quantification of Wnt4 protein levels in TEAD1^fl/fl^ and TEAD1^fl/fl^postn^+^ mice after sham or TAC surgery for 4 weeks (n=4 per group). **c.** Western blot and quantification of Wnt4 protein levels in TEAD1^fl/fl^ and TEAD1^fl/fl^col1a2^+^ mice infused with saline or Ang-II for 4 weeks (n=4 per group). **d.** Western blot and quantification of Wnt4 protein levels in TEAD1^fl/fl^ and TEAD1^fl/fl^postn^+^ mice infused with saline or Ang-II for 4 weeks (n=4 per group). For all statistical plots, the data are presented as mean ± SD. ns. indicates no significance between the 2 indicated groups. a-d by two-way ANOVA with Bonferroni multiple comparison test.


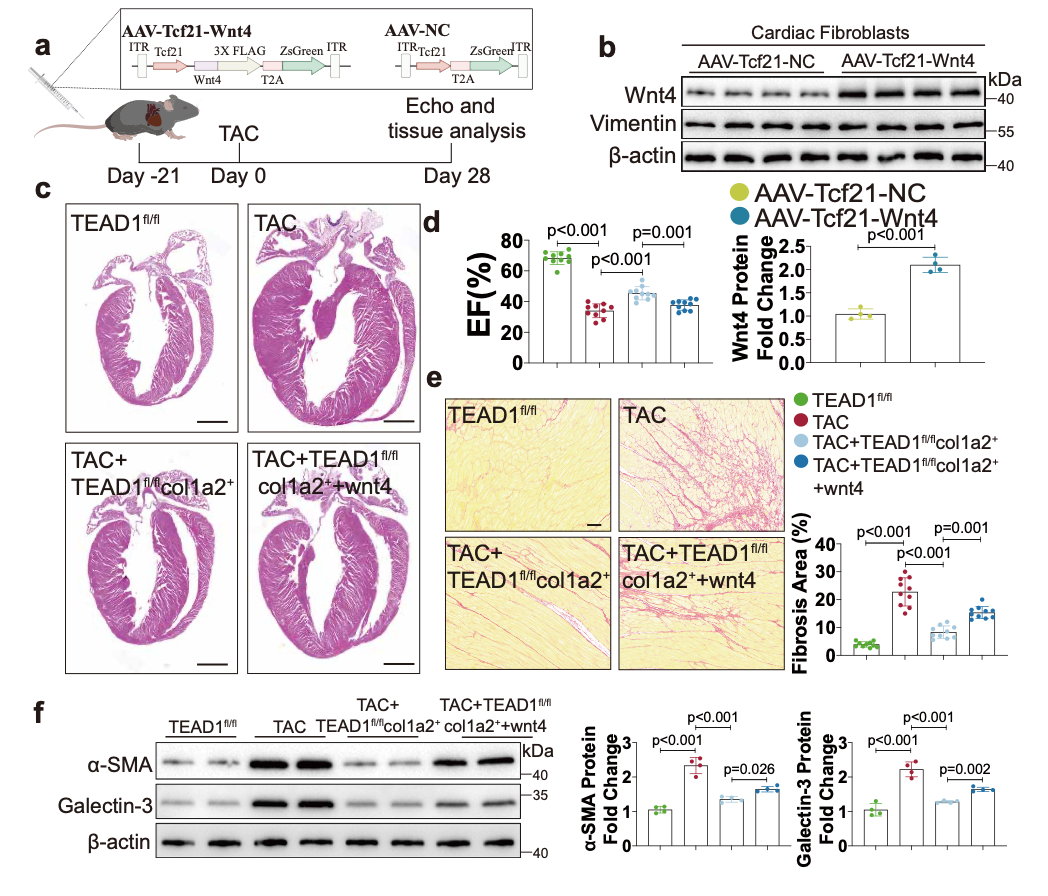


**Figure. S12. Wnt4 overexpression blocked the protection effect of TEAD1 knock out in CFs population.**

**a.** Schematic illustration of Wnt4 overexpression in vivo. **b.** Western blot and quantification of Wnt4 and Vimentin protein levels in CFs isolated from mice subjected to AAV9-Tcf21-NC or AAV9-Tcf21-Wnt4 administration (n=4 per group). **c.** Heart sections from mice of indicated groups were stained with hematoxylin and eosin to show whole-heart gross images (n=10 per group; scale bar=5mm). **d-e.** Left ventricular EF (**d**) and FS (**e**) assessed by echocardiography of mice from indicated groups (n=10 per group). **f.** Heart sections from mice of indicated groups were stained with picrosirius red to visualize collagen deposition (n=10 per group; scale bar=50um). **g.** Western blot and quantification of α‑SMA and galectin-3 protein levels in the heart homogenates extracted from mice of indicated groups (n=4 per group). For all statistical plots, the data are presented as mean ± SD. b by two-tailed unpaired Student’s t-test; d-g by one-way ANOVA with Bonferroni multiple comparison test.

**Figure. S13. YAP is involved in the TEAD1-BRD4 complex.**

**a.** Western blot and quantification of Wnt4 protein levels in CFs transfected with si-NC or si-YAP and then treated with saline or Ang-II for 48 hours (n=4 per group). **b-c.** Endogenous immunoprecipitation of YAP, TEAD1 and BRD4 in CFs in the presence or absence of VT103. **d.** Western blot and quantification of p-YAP and YAP protein levels in CFs stimulated with saline or Ang-II in the presence or absence of VT103 (n=4 per group). For all statistical plots, the data are presented as mean ± SD. a by two-way ANOVA with Bonferroni multiple comparison test. d by one-way ANOVA with Bonferroni multiple comparison test.

**Figure. S14. TEAD1 activates Wnt signaling pathway via interaction with BRD4.**

**a.** qRT-PCR analyses of the mRNA levels of Wnt4 and ACTA2 in CFs infected with adenovirus expressing TEAD1 or NC in the presence or absence of JQ1 (n=4 per group). **b.** qRT-PCR analyses of the mRNA levels of Wnt4 and ACTA2 in CFs infected with adenovirus expressing TEAD1 or BRD4 (n=4 per group). **c.** Western blot of TEAD1, BRD4, Wnt4, α‑SMA and Galectin-3 protein levels in CFs infected with adenovirus expressing TEAD1 or BRD4 (n=4 per group). **d.** Representative images of immunofluorescence staining against α-SMA and quantification of the CFs infected with adenovirus expressing TEAD1 or NC in the presence or absence of JQ1 (n=3 per group, scale bar=100 μm). **e.** Migration of CFs infected with adenovirus expressing TEAD1 or NC in the presence or absence of JQ1 (n=3 per group; scale bar=100 μm). **f.** Collagen gel contraction seeded CFs infected with adenovirus expressing TEAD1 or NC in the presence or absence of JQ1 (n=3 per group). For all statistical plots, the data are presented as mean ± SD. ns. indicates no significance between the 2 indicated groups. a and b by two-way ANOVA with Bonferroni multiple comparison test. c-f by one-way ANOVA with Bonferroni multiple comparison test.

**Figure. S15. Safety evaluation of TEAD1 inhibitor VT103.**

**a-c**. Concentration of aspartate aminotransferase (AST) and alanine aminotransferase (ALT), lactate dehydrogenase (LDH) and creatine kinase (CK), creatinine in the serum from mice treated with VT103 versus vehicle (n=10 per group). **d, e**. Body weight and heart rate of mice treated with VT103 versus vehicle (n=10 per group). **f, g**. Hematoxylin and eosin staining of liver sections and kidney sections from mice treated with VT103 versus vehicle. For all statistical plots, the data are presented as mean ± SD. ns. indicates no significance between the 2 indicated groups. a-e by two-tailed unpaired Student’s t-test.

**Table S1.** Table of sequences and downstream primers for genes analyzed by qRT-PCR(h-human, m-mouse).

| Gene | Forward Primer | Reverse Primer |
| --- | --- | --- |
| TEAD1(h) | 5’-aaggtcgctccattggcacaac-3’ | 5’-atgggtcactgtaagaatggttggc-3’ |
| β-actin (h) | 5’-aggattcctatgtgggcgac -3’ | 5’-atagcacagcctggatagcaa -3’ |
| TEAD1(m) | 5’-gatgagcgactcggcagataagc -3’ | 5’- tcccacacggcggatagatagc -3’ |
| β-MHC(m) | 5’-caccagcctcatcaaccagaagaag-3’ | 5’-tcctctgcgttcctacactcctg-3’ |
| BNP(m) | 5’-ggaagtcctagccagtctccagag-3’ | 5’-gccttggtccttcaagagctgtc-3’ |
| ANP(m) | 5’-aagaacctgctagaccacctggag-3’ | 5’-tgcttcctcagtctgctcactcag-3’ |
| Wnt4 (m) | 5’-gcgtagccttctcacagtcctttg-3’ | 5’-cttcctgccagcctcgttgttg -3’ |
| ACTA2 (m) | 5’-ccttcgtgactactgccgag-3’ | 5’-ataggtggtttcgtggatgc -3’ |
| β-actin (m) | 5’-gtgacgttgacatccgtaaaga-3’ | 5’- gtgacgttgacatccgtaaaga-3’ |

**Table S2.** Clinical characteristics of patients with Non-HCM or HCM.

| Non-HCM (n=8) HCM (n=10) p |
| --- |
| Age of diagnosis (years) 54.52±5.57 61.56±3.56 0.005  Female n (%) 4 (50%) 4 (40%) >0.999  Family history of HCM (%) 1 (12.5%) 5 (50%) 0.152  BMI 23.64±2.26 25.31±2.67 0.178 |
| Echocardiographic parameters  LVEDD (mm) 41.01±3.54 63.06±4.54 <0.001  LVESD (mm) 35.01±3.48 42.01±6.28 0.012  LVEF (%) 54.36 ± 4.23 28 ± 3.56 <0.001 |
| Diseases  Hypertension (%) 5 (62.5%) 4 (40%) 0.637  Atrial fibrillation (%) 2 (25%) 2 (20%) >0.999  Valvular heart disease (%) 2 (25%) 1 (10%) 0.559  Diabetes (%) 2 (25%) 3 (30%) >0.999  Coronary diseases (%) 2 (25%) 3 (30%) >0.999  Obstructive HCM 0 4 (40%) 0.092  Nonobstructive HCM 0 6 (60%) 0.013  Medical therapy prior to the surgery  Anticoagulant n (%) 1 (12.5%) 3 (30%) 0.588  Antiplatelet n (%) 4 (50%) 7 (70%) 0.631  Statin n (%) 3 (37.5%) 8 (80%) 0.145  Calcium antagonist n (%) 1 (12.5%) 7 (70%) 0.026  Diuretics n (%) 2 (25%) 10 (100%) 0.002  β-blockers n (%) 4 (50%) 9 (90%) 0.118  ACEI/ARB n (%) 4 (50%) 9 (90%) 0.118 |
| Data are expressed as mean ± SD. Data of Ages, BMI and Echocardiographic parameters were analyzed using two-tailed unpaired Student’s t-test. Others were analyzed using Fisher exact test. HCM hypertrophic cardiomyopathy, BMI body mass index, LVEDD left ventricular end diastolic diameter, LVESD left ventricular end systolic diameter, LVEF left ventricular ejection fraction, ACEI angiotensin-converting enzyme inhibitor, and ARB angiotensin receptor blocker |

**Table S3.** Biometric and cardiac parameters of TEAD1^fl/fl^ and TEAD1^fl/fl^col1a2^+^ mice on days 28 post-TAC or Sham.

| Group | TEAD1^fl/fl^Sham | TEAD1^fl/fl^col1a2^+^ | TEAD1^fl/fl^ TAC | TEAD1^fl/fl^col1a2^+^  TAC |
| --- | --- | --- | --- | --- |
| n | 4 | 10 | 10 | 10 |
| BW (g) | 22.35±0.43 | 23.54±0.33 | 23.06±0.41 | 22.86±0.62 |
| HR (beats/min) | 513.3±14.1 | 531.5±18.2 | 506.7±20.1 | 515.7±15.0 |
| LVID_d_ | 3.737±0.178 | 3.638±0.158 | 4.675±0.317^***^ | 4.103±0.254**^###^** |
| LVID_s_ | 2.494±0.241 | 2.518±0.219 | 3.914±0.237^***^ | 3.231±0.216**^###^** |
| IVS_d_ | 0.800±0.082 | 0.815±0.073 | 1.053±0.118^**^ | 0.913±0.109**^#^** |
| IVS_s_ | 1.208±0.100 | 1.214±0.114 | 1.416±0.137^***^ | 1.284±0.128**^###^** |
| LVPW_d_ | 0.731±0.049 | 0.742±0.042 | 1.043±0.123^***^ | 0.816±0.107**^##^** |
| LVPW_s_ | 1.034±0.075 | 1.062±0.058 | 1.274±0.141^***^ | 1.101±0.108**^##^** |
| EF (%) | 66.3±5.0 | 67.9±5.8 | 32.1±4.6^***^ | 42.8±2.8**^###^** |
| FS (%) | 34.4±2.6 | 35.3±3.0 | 16.4±2.3^***^ | 22.4±1.8**^###^** |
| HW/BW (mg/g) | 4.29±0.19 | 4.57±0.22 | 7.89±1.05^***^ | 5.66±0.47**^###^** |
| HW/TL(mg/mm) | 6.27±0.23 | 6.33±0.64 | 11.51±1.18^***^ | 8.85±0.86**^###^** |

Data are expressed as mean ± SD. Data was analyzed using two-way ANOVA followed by Bonferroni post hoc analysis. ***p<0.001 vs. TEAD1^fl/fl^Sham; **^###^**p<0.001 vs. TEAD1^fl/fl^TAC. BW, body weight; HR, heart rate; EF, ejection fraction; FS, fractional shortening; HW/BW, heart weight /body weight; HW/TL, heart weight/tibia length.

**Table S4.** Biometric and cardiac parameters of TEAD1^fl/fl^ and TEAD1^fl/fl^col1a2^+^ mice on days 28 post-Ang-II or Saline.

| Group | TEAD1^fl/fl^Saline | TEAD1^fl/fl^col1a2^+^ | TEAD1^fl/fl^  Ang-II | TEAD1^fl/fl^col1a2^+^  Ang-II |
| --- | --- | --- | --- | --- |
| n | 4 | 10 | 10 | 10 |
| BW (g) | 22.85±0.31 | 23.24±0.38 | 22.36±0.42 | 23.12±0.51 |
| HR (beats/min) | 525.3±9.3 | 514.1±11.4 | 515.7±18.1 | 506.7±12.4 |
| LVID_d_ | 3.638±0.185 | 3.716±0.173 | 3.343±0.218 | 3.276±0.195 |
| LVID_s_ | 2.437±0.227 | 2.562±0.256 | 2.054±0.183 | 2.032±0.183 |
| IVS_d_ | 0.806±0.080 | 0.823±0.063 | 1.345±0.131^***^ | 1.045±0.105**^###^** |
| IVS_s_ | 1.223±0.107 | 1.300±0.121 | 1.521±0.129^***^ | 1.343±0.132**^###^** |
| LVPW_d_ | 0.742±0.052 | 0.775±0.039 | 1.214±0.154^***^ | 0.953±0.126**^###^** |
| LVPW_s_ | 1.026±0.080 | 1.034±0.069 | 1.505±0.138^***^ | 1.252±0.130**^###^** |
| EF (%) | 61.3±3.0 | 64.8±5.0 | 65.2±4.4 | 65.2±3.9 |
| FS (%) | 31.6±1.3 | 33.2±2.8 | 33.2±2.8 | 33.0±2.1 |
| HW/BW(mg/g) | 4.27±0.13 | 4.71±0.34 | 8.00±0.73^***^ | 5.83±0.57**^###^** |
| HW/TL(mg/mm) | 6.08±0.16 | 6.65±0.39 | 12.69±1.97^***^ | 9.35±1.35**^###^** |

Data are expressed as mean ± SD. Data was analyzed using two-way ANOVA followed by Bonferroni post hoc analysis. ***p<0.001 vs. TEAD1^fl/fl^Saline; **^###^**p<0.001 vs. TEAD1^fl/fl^ Ang-II. BW, body weight; HR, heart rate; EF, ejection fraction; FS, fractional shortening; HW/BW, heart weight /body weight; HW/TL, heart weight/tibia length.

**Table S5.** Biometric and cardiac parameters of TEAD1^fl/fl^ and TEAD1^fl/fl^postn^+^ mice on days 28 post-TAC or Sham.

| Group | TEAD1^fl/fl^  Sham | TEAD1^fl/fl^postn^+^ | TEAD1^fl/fl^ TAC | TEAD1^fl/fl^postn^+^  TAC |
| --- | --- | --- | --- | --- |
| n | 4 | 10 | 10 | 10 |
| BW (g) | 23.15±0.37 | 22.64±0.28 | 23.11±0.37 | 23.06±0.54 |
| HR (beats/min) | 530.5±8.0 | 510.7±11.4 | 508.8±12.3 | 520±13.3 |
| LVID_d_ | 3.674±0.169 | 3.757±0.148 | 4.721±0.307^***^ | 4.210±0.278**^###^** |
| LVID_s_ | 2.389±0.252 | 2.416±0.273 | 3.835±0.251^***^ | 3.156±0.253**^###^** |
| IVS_d_ | 0.821±0.068 | 0.818±0.058 | 1.069±0.125^**^ | 0.923±0.113**^#^** |
| IVS_s_ | 1.198±0.108 | 1.201±0.110 | 1.432±0.127^**^ | 1.237±0.131**^##^** |
| LVPW_d_ | 0.764±0.042 | 0.756±0.039 | 1.031±0.135^**^ | 0.943±0.116**^#^** |
| LVPW_s_ | 1.046±0.068 | 1.052±0.059 | 1.258±0.134^**^ | 1.104±0.113**^#^** |
| EF (%) | 70.8±3.6 | 70.7±5.7 | 31.0±3.7^***^ | 45.9±7.00**^###^** |
| FS (%) | 36.4±1.8 | 36.4±3.2 | 16.1±2.1^***^ | 24.3±3.6**^###^** |
| HW/BW(mg/g) | 4.54±0.09 | 4.66±0.22 | 7.47±0.96^***^ | 5.54±0.58**^###^** |
| HW/TL(mg/mm) | 6.20±0.70 | 6.34±0.64 | 11.91±1.62^***^ | 8.61±1.11**^###^** |

Data are expressed as mean ± SD. Data was analyzed using two-way ANOVA followed by Bonferroni post hoc analysis. ***p<0.001 vs. TEAD1^fl/fl^ Sham; **^###^**p<0.001 vs. TEAD1^fl/fl^ TAC. BW, body weight; HR, heart rate; EF, ejection fraction; FS, fractional shortening; HW/BW, heart weight /body weight; HW/TL, heart weight/tibia length.

**Table S6.** Biometric and cardiac parameters of TEAD1^fl/fl^ and TEAD1^fl/fl^postn^+^ mice on days 28 post-Ang-II or Saline.

| Group | TEAD1^fl/fl^ Saline | TEAD1^fl/fl^postn^+^ | TEAD1^fl/fl^  Ang-II | TEAD1^fl/fl^postn^+^  Ang-II |
| --- | --- | --- | --- | --- |
| n | 4 | 10 | 10 | 10 |
| BW (g) | 22.74±0.31 | 23.31±0.22 | 23.43±0.28 | 23.31±0.57 |
| HR (beats/min) | 503.5±12.4 | 512.3±9.6 | 502.5±12.9 | 520.1±15.3 |
| LVID_d_ | 3.729±0.146 | 3.776±0.139 | 3.241±0.187 | 3.174±0.128 |
| LVID_s_ | 2.376±0.186 | 2.403±0.216 | 2.021±0.201 | 2.132±0.165 |
| IVS_d_ | 0.812±0.076 | 0.788±0.059 | 1.401±0.142^***^ | 1.115±0.138**^###^** |
| IVS_s_ | 1.187±0.110 | 1.216±0.102 | 1.535±0.126^***^ | 1.256±0.210**^###^** |
| LVPW_d_ | 0.729±0.043 | 0.759±0.051 | 1.312±0.138^***^ | 0.954±0.174**^###^** |
| LVPW_s_ | 1.036±0.074 | 1.041±0.053 | 1.499±0.142^***^ | 1.215±0.163**^###^** |
| EF (%) | 64.8±2.5 | 65.9±2.4 | 63.9±1.7 | 64.4±3.5 |
| FS (%) | 33.4±1.3 | 34.0±1.2 | 33.4±1.3 | 33.2±2.4 |
| HW/BW(mg/g) | 4.44±0.14 | 4.74±0.32 | 7.76±0.72^***^ | 5.79±0.44**^###^** |
| HW/TL(mg/mm) | 6.39±0.38 | 6.46±0.42 | 12.64±1.48^***^ | 8.75±0.82**^###^** |

Data are expressed as mean ± SD. Data was analyzed using two-way ANOVA followed by Bonferroni post hoc analysis. ***p<0.001 vs. TEAD1^fl/fl^Saline; **^###^**p<0.001 vs. TEAD1^fl/fl^Ang-II. BW, body weight; HR, heart rate; EF, ejection fraction; FS, fractional shortening; HW/BW, heart weight /body weight; HW/TL, heart weight/tibia length.

**Table S7.** Biometric and cardiac parameters of Vehicle and VT103 mice on days 28 post-TAC or Sham.

| Group | Sham+Vehicle | Sham+VT103 | TAC+Vehicle | TAC+VT103 |
| --- | --- | --- | --- | --- |
| n | 4 | 10 | 10 | 10 |
| BW (g) | 23.21±0.45 | 22.61±0.38 | 23.21±0.42 | 23.54±0.33 |
| HR (beats/min) | 507.6±12.6 | 498.8±13.2 | 525.5±9.6 | 515.6±14.2 |
| LVID_d_ | 3.693±0.231 | 3.658±0.195 | 4.734±0.267^***^ | 4.200±0.179**^###^** |
| LVID_s_ | 2.506±0.189 | 2.467±0.216 | 3.834±0.231^***^ | 3.178±0.301**^###^** |
| IVS_d_ | 0.786±0.068 | 0.807±0.058 | 1.174±0.128^**^ | 0.936±0.111**^##^** |
| IVS_s_ | 1.234±0.107 | 1.265±0.101 | 1.466±0.126^**^ | 1.298±0.106**^##^** |
| LVPW_d_ | 0.746±0.038 | 0.783±0.036 | 1.064±0.141^***^ | 0.912±0.114**^#^** |
| LVPW_s_ | 1.067±0.058 | 1.044±0.048 | 1.267±0.127^***^ | 1.145±0.117**^#^** |
| EF (%) | 66.5±5.2 | 68.3±6.7 | 33.8±6.3^***^ | 44.5±5.6**^##^** |
| FS (%) | 33.8±3.0 | 34.0±3.4 | 18.3±3.0^***^ | 23.0±2.8**^##^** |
| HW/BW(mg/g) | 4.59±0.58 | 4.82±0.41 | 7.96±1.30^***^ | 5.98±0.53**^###^** |
| HW/TL(mg/mm) | 6.56±0.44 | 6.33±0.38 | 11.38±1.57^***^ | 9.30±0.96**^###^** |

Data are expressed as mean ± SD. Data was analyzed using two-way ANOVA followed by Bonferroni post hoc analysis. ***p<0.001 vs. Sham+Vehicle; **^###^**p<0.001 vs. TAC+Vehicle. BW, body weight; HR, heart rate; EF, ejection fraction; FS, fractional shortening; HW/BW, heart weight /body weight; HW/TL, heart weight/tibia length.

**Table S8.** The information of antibodies.

| Name | Vendor | Cat |
| --- | --- | --- |
| TEAD1 | Abcam | Ab133533 |
| TEAD1 | Invitrogen | GT13112 |
| TEAD1 | CST | 12292 |
| α-SMA | Abcam | Ab124964 |
| Ig G | CST | 2729 |
| β-actin | CST | 4970 |
| Galectin-3 | Abclonal | A11198 |
| Collagen III | SantaCruz | Sc271249 |
| Collagen I | Abcam | ab260043 |
| wnt4 | Invitrogen | PA527321 |
| wnt4 | Abcam | ab262696 |
| β-Catenin | Abcam | ab32572 |
| Histone H3 | CST | 4620 |
| BRD4 | Abcam | ab128874 |
| BRD4 | CST | 63759 |
| PKC | Abcam | ab32376 |
| p-JNK | Abcam | ab124956 |
| SAPK/JNK | Abcam | ab9252 |
| Vimentin | Abcam | ab8978 |
| CaMKII | Abcam | ab134041 |
| YAP | CST | 14074 |
| HRP-labeled Goat Anti-Rabbit IgG(H+L) | Beyotime | A0208 |
| Goat anti-Rabbit IgG (H+L) Alexa Fluor Plus 488 | invitrogen | A32731 |
| Goat anti-Rabbit IgG (H+L) Alexa Fluor Plus 647 | invitrogen | A32733 |
| Goat anti-mouse IgG (H+L) Alexa Fluor Plus 488 | invitrogen | A32766 |
| Goat anti-Rabbit IgG (H+L) Alexa Fluor Plus 647 | invitrogen | A32733 |

**Reference:**

1 Zheng, B., Zhang, Z., Black, C. M., de Crombrugghe, B. & Denton, C. P. Ligand-dependent genetic recombination in fibroblasts : a potentially powerful technique for investigating gene function in fibrosis. *Am J Pathol.* **160**, 1609-1617 (2002).

2 Kanisicak, O. et al. Genetic lineage tracing defines myofibroblast origin and function in the injured heart. *Nat Commun.* **7**, 12260 (2016).

3 Song, S. et al. Inhibition of BRD4 attenuates transverse aortic constriction- and TGF-beta-induced endothelial-mesenchymal transition and cardiac fibrosis. *J Mol Cell Cardiol.* **127**, 83-96 (2019).

4 Song, S. et al. EZH2 as a novel therapeutic target for atrial fibrosis and atrial fibrillation. *J Mol Cell Cardiol.* **135**, 119-133 (2019).

5 Pan, Z. et al. MicroRNA-101 inhibited postinfarct cardiac fibrosis and improved left ventricular compliance via the FBJ osteosarcoma oncogene/transforming growth factor-beta1 pathway. *Circulation.* **126**, 840-850 (2012).

6 Ackers-Johnson, M. et al. A Simplified, Langendorff-Free Method for Concomitant Isolation of Viable Cardiac Myocytes and Nonmyocytes From the Adult Mouse Heart. *Circ Res.* **119**, 909-920 (2016).

7 Chen, J. et al. VEGF amplifies transcription through ETS1 acetylation to enable angiogenesis. *Nat Commun.* **8**, 383 (2017).

8 Wang, Q. et al. Disulfiram bolsters T-cell anti-tumor immunity through direct activation of LCK-mediated TCR signaling. *EMBO J.* **41**, e110636 (2022).
